# Reactive oxygen species reprogram macrophages to suppress antitumor immune response through the exosomal miR-155-5p/PD-L1 pathway

**DOI:** 10.1186/s13046-022-02244-1

**Published:** 2022-01-27

**Authors:** Xiang Li, Shaomin Wang, Wei Mu, Jennifer Barry, Anna Han, Richard L. Carpenter, Bing-Hua Jiang, Stephen C. Peiper, Mỹ G. Mahoney, Andrew E. Aplin, Hong Ren, Jun He

**Affiliations:** 1 Department of Pathology, Anatomy & Cell Biology, Sidney Kimmel Cancer Center, Thomas Jefferson University, Philadelphia, USA; 2 Department of Otorhinolaryngology-Head and Neck Surgery, The First Affiliated Hospital of Xi’an Jiaotong University, Xi’an, Shaanxi 710061 P. R. China; 3 School of Public Health, Shanghai Jiaotong University School of Medicine, Shanghai, China; 4 Department of Cancer Biology, Sidney Kimmel Cancer Center, Thomas Jefferson University, Philadelphia, USA; 5 Department of Biochemistry and Molecular Biology, Indiana University School of Medicine, Bloomington, IN 47405 USA; 6 Department of Dermatology and Cutaneous Biology, Thomas Jefferson University, Philadelphia, USA; 7 Department of Thoracic Surgery and Oncology, Cancer Center, The First Affiliated Hospital of Xi’an Jiaotong University, Xi’an, Shaanxi 710061 P. R. China

**Keywords:** Reactive oxygen species, Tumor immune response, Ovarian cancer, Tumor-associated macrophages, Tumor exosomes, miR-155-5p, PD-L1

## Abstract

**Background:**

Cancer cells have an imbalance in oxidation-reduction (redox) homeostasis. Understanding the precise mechanisms and the impact of the altered redox microenvironment on the immunologic reaction to tumors is limited.

**Methods:**

We isolated exosomes from ovarian cancer cells through ultracentrifuge and characterized by Western-blots and Nanoparticle Tracking Analysis. 2D, 3D-coculture tumor model, and 3D live cell imaging were used to study the interactions between tumor cells, macrophages and CD3 T cells in vitro. The role of exosomal miR-155-5p in tumor growth was evaluated in xenograft nude mice models and immune-competent mice models. Flow cytometry and flow sorting were used to determine the expression levels of miR-155-5p and PD-L1 in ascites and splenic macrophages, and the percentages of CD3 T cells subpopulations.

**Results:**

The elevation of reactive oxygen species (ROS) greatly downregulated exosomal miR-155-5p expression in tumor cells. Neutralization of ROS with N-acetyl-L-cysteine (NAC) increased the levels of miR-155-5p in tumor exosomes that were taken up by macrophages, leading to reduction of macrophage migration and tumor spheroid infiltration. We further found that programmed death ligand 1 (PD-L1) is a functional target of miR-155-5p. Co-culture of macrophages pre-treated with NAC-derived tumor exosomes or exosomal miR-155-5p with T-lymphocytes leading to an increased percentage of CD8^+^ T-lymphocyte and a decreased CD3^+^ T cell apoptosis through PD-L1 downregulation. Tumor growth in nude mice was delayed by treatment with NAC-derived tumor exosomes. Delivery of tumor exo-miR-155-5p in immune-intact mice suppressed ovarian cancer progression and macrophage infiltration, and activated CD8^+^ T cell function. It is of note that exo-miR-155-5p inhibited tumor growth more potently than the PD-L1 antibody, suggesting that in addition to PD-L1, other pathways may also be targeted by this approach.

**Conclusions:**

Our findings demonstrate a novel mechanism, ROS-induced down-regulation of miR-155-5p, by which tumors modulate the microenvironment that favors tumor growth. Understanding of the negative impact of ROS on the tumor immune response will improve current therapeutic strategies. Targeting miR-155-5p can be an alternative approach to prevent formation of an immunosuppressive TME through downregulation of PD-L1 and other immunosuppressive factors.

**Graphical Abstract:**

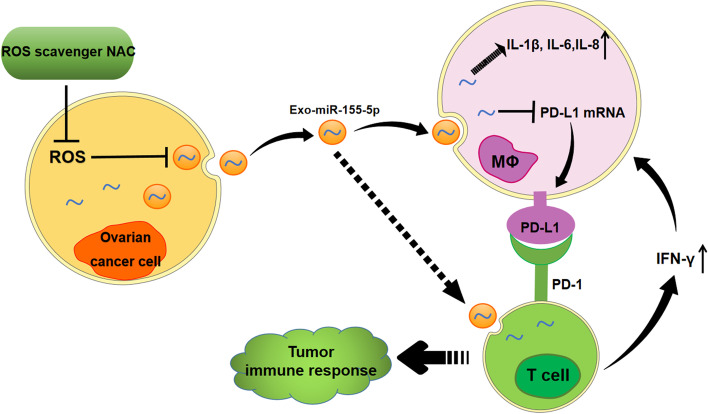

**Supplementary Information:**

The online version contains supplementary material available at 10.1186/s13046-022-02244-1.

## Background

Epithelial ovarian cancer (EOC) is the most lethal gynecological cancer in the United States, with an estimated 22,530 new cases diagnosed and 13,980 deaths in 2019 [1, 2]. Despite the standard first-line chemotherapy following cytoreductive surgery, the mortality rate has not improved in the era of targeted-therapy; the 5-year survival rate is around 39% for all EOC and 30% for in patients at stage III and IV [1]. More than half of cases are diagnosed at advanced stages with extensive intraperitoneal disseminated metastasis and chemotherapeutic resistance [3, 4]. Growing evidence suggests that the peritoneal tumor microenvironment (TME) plays an essential role in ovarian cancer progression, metastasis, and the development of drug resistance [5]. During metastasis of ovarian cancer, the intraperitoneal tumor microenvironment forms an immunosuppressive milieu with accumulated ascites that contain a large number of tumor spheroids and various stromal and immune cells, such as macrophages and lymphocytes, as well as soluble pro-tumor mediators [6]. The macrophages that infiltrate into the tumor microenvironment are defined as tumor-associated macrophage (TAMs), which generally display an anti-inflammatory and pro-tumor M2-like phenotype, and thus play an important role in facilitating the peritoneal dissemination of ovarian cancer cells [7]. Increased recruitment of TAMs into tumor spheroids floating in the ascites has been positively correlated with poor outcomes in patients with advanced ovarian cancer [6]. It remains unclear how ovarian tumor cells evade immune surveillance, a key obstacle to develop effective immunotherapy for ovarian cancer treatment.

Exosomes are nanometer-sized membrane encapsulated vesicles released by all cell types to convey information to neighboring or distant cells by transporting cytosolic biomolecules, such as proteins, DNA, mRNAs, and miRNAs, and thus influence recipient cells [8, 9]. Among all of the cell types, tumor cells secrete at least 10-fold more exosomes than other cells [10]. Exosomes generated by tumors carry cargos that partially mimic parent cell content, and thereby they reprogram recipient cells into active contributors to angiogenesis, metastasis, and immunosuppression [11]. Tumor cells generate distinct exosomes that are regulated by external signals, particularly from oxidative stress [12]. We previously showed that ovarian cancer cells produce excessive reactive oxygen species (ROS) with NOX4 being the major contributor [13]. ROS can regulate the biogenesis and expression of miRNAs, which may in turn affect redox signaling pathways and thus promote tumorigenesis and progression [14, 15]. However, it has yet to be determined how these miRNAs convey the ROS signal to downstream effectors leading to alterations in the extracellular milieu that creates a redox microenvironment. The current study aimed to investigate whether and how ROS influence the TME to promote ovarian cancer development through tumor exosomal miRNAs. We found that ROS decreased the amount of tumor exo-miR-155 that was taken up by macrophages, resulting in enhanced macrophage infiltration and T cell inactivation characterized by upregulation of programmed death ligand 1 (PD-L1). Targeting ROS/miR-155-5p is a promising strategy to prevent the formation of the suppressive tumor microenvironment in ovarian cancer.

## Methods

### Cell lines, reagents, and antibodies

Human ovarian cancer cell line A2780, Ovcar-3, and SKOV-3, murine ovarian cancer cell line ID8, and human peripheral blood monocytes THP-1 were purchased from the American Type Culture Collection (ATCC). Peripheral blood mononuclear cells (PBMC) were purchased from Stemcell Technologies. All cell lines were cultured in RPMI 1640 or in Dulbecco’s modified Eagle’s (DMEM) media supplemented with 10% FBS. All cell lines did not have mycoplasma contamination determined by RT-PCR. N-acetyl-L-cysteine (NAC) was purchased from Selleckchem, and phorbol 12-myristate 13-acetate (PMA), catalase-PEG, and rotenone were from Sigma Aldrich. The sources of antibodies used in immunoblotting, flow cytometry, and IHC were shown in Suppl. Table 2.

### Exosomes purification from cell culture supernatant, ascites, and serum

Exosomes from cell culture medium were isolated by ultracentrifugation. Ovarian cancer cells were grown in T75 flasks until they reached to 70% confluency, and then were rinsed with PBS and then replaced with serum-free medium for 48 h. The supernatant was collected and centrifuged at 2000×g for 30 min to discard cellular debris. Next, the collected medium were centrifuged at 12000×g for 30 min at 4 °C followed by filtration through 0.22-μm pore filters (Steriflip, Millipore) to remove larger extracellular vesicles. The supernatant were then and ultracentrifuged at 110,000×g for 2 h at 4 °C (Beckman Coulter, Ti45 rotor). The exosomes pellets were re-suspended in 10 ml PBS, and loaded over 10 ml of 40% sucrose solution, and was ultracentrifuged at 110,000×g for 90 min. The lower sucrose layer, which contained the exosomes was diluted with PBS and ultracentrifuged at 110,000×g for 90 min. The pellet was resuspended in 100 μl PBS and stored in − 80 °C. Exosomes from ascites in mice were isolated using the same method except for erythrocytes lysis with ACK lysis buffer (Thermo Fisher Scientific) after the initial centrifuge at 1500×g for 15 min to remove debris. Exosomes from the serum in mice were isolated using Exosome Isolation Reagent (Invitrogen, Thermo Fisher Scientific). The serum exosomes were characterized by NTA.

### Nanoparticle tracking analysis (NTA)

NTA was performed using a NanoSight NS300 instrument according to the manufacturer’s instructions. Isolated exosome samples were diluted with PBS at 1:100 before analysis. The settings after NTA collection were optimized and remained constant between samples. Three videos of 60 s length were recorded for each sample and analyzed to give estimates of mean size and number of particles.

### Nanostring nCounter miRNA expression assay

All samples were prepared and processed according to NanoString nCounter Expression CodeSet Design Manual. 100 ng of exosomal RNAs were used as input for Nanostring nCounter miRNA sample preparation. Raw data were normalized to the top 100 miRs using nSolver analysis 2.5 software (Nanostring). Expression heatmaps were generated with the R package pheatmap.

### Fluorescence in situ hybridization

In situ hybridization was performed using double digoxigenin (DIG)-labeled miRCURY LNA miRNA detection probe hsa-miR-155-5p (Advanced Cell Diagnostics, CA, USA) in human ovarian carcinoma tissue microarray (US Biomax, MD, USA). The TMA sections contain 160 cases of ovarian cancer tissue sections and 40 cases of normal human ovary tissue sections. The experiments were performed according to the miRNAscope LS Automated Assay (ACD, CA, USA) protocol. The signal was detected with miRNAscope™ LS Reagent Kit - RED (ACD, CA, USA). Slow Fade Gold anti-fade reagent with DAPI (Life Technologies) was employed as the mounting medium. The positive and negative control of the method was verified employing miRNAscope LS Positive Control Probe (ACD, CA, USA) and miRNAscope LS Negative Control Probe (ACD, CA, USA), respectively. Bright field pictures were scanned by Pannoramic slide scanner (3DHISTECH) and converted to fluorescence signal with Caseviewer 2.2 software. Pictures were taken by Image-Pro Plus 6.0 software. Slides were analyzed with Quant Center 2.1 (Thermo Fisher Scientific) and Graphpad Prism software (La Jolla, CA, USA).

### RT-qPCR

Total RNAs were extracted using Trizol (Thermo Fisher Scientific). The cDNA synthesis was performed using oligo (dT)18 primers and M-MLV reverse transcriptase. The 100 ng of RT product was used for PCR reaction using Power SYBR Green PCR Master Mix (Thermo Fisher Sci). The primer sequences are listed in Supplementary Table 1.

Two-step Taqman-qPCR analysis was performed to assess miRNA levels using Taqman miRNA reverse transcription kit and Taqman universal PCR master mix (Thermo Fisher Sci) in accordance with manufacturer’s instructions.

### Fluorescent labeling of exosomes with DiR

Exosomes were fluorescently labeled using lipophilic carbocyanine DiR dye (Thermo Fisher Scientific). In brief, 5 μl of DiR (200 μg/ml) in ethanol was mixed with 200 μg exosomes in 100 μl PBS for 1 h. The unincorportated DiR was removed by a Sepharose CL-4B column (Sigma-Aldrich).

### Dual luciferase reporter assay

THP-1 cells were seeded in 12-well plates at a number of 5 × 10^5^ and were co-transfected with PD-L1-WT 3′-UTR reporter plasmids (Addgene) and miR-cont, miR-155-5p mimics, miR-152-3p mimics, or miR-137 mimics, using jetPRIME® reagent. The transfected cells were harvested 48 h post-transfection using Passive Lysis Buffer (Promega, US), and firefly and Renilla luciferase activities were measured in cell lysates using Dual-Luciferase Reporter Assay System (Promega).

### 3D-coculture tumor model and 3D live cell imaging

A2780-miR-con and A2780-miR-155 cells were seeded onto 1% agarose (Sigma-Aldrich) substrate in the cover glass bottom of 48-well plates (MatTek, MA, USA) at 1 × 10^4^ cells/well. After 4 days of formation of tumor spheroids, macrophages were added and allowed to co-culture with tumor spheroids for an additional 4 days. The diameters of spheroids were measured at 3 d, 7 d, 10 d and 12 d, respectively. For the 3D live cell imaging, the cells were stained with Cell tracker green (Thermo Fisher Scientific) before seeding onto 1% agarose concave surface for spheroid formation. The infiltrated macrophages were stained by eFluor670 (Thermo Fisher Scientific) before adding into tumor spheroids. The pictures of the 3D co-culture were taken by an A1R+ Nikon confocal microscope for 3D live cell imaging. The number of A2780 and infiltrated macrophages were presented as accumulative average intensity of fluorescence. Z stack-overlay video was taken by A1R+ Nikon confocal microscope.

### Flow cytometry and fluorescence-activated cell sorting (FACS)

For cultured cells or 3D co-culture cells, single-cell suspensions were incubated with 2% human Fc Receptor block (eBioscience, Frankfurt, Germany) in PBS for 20 min on ice. After washing with PBS, cells were stained with conjugated antibodies for 30 min at room temperature in the dark. For tumors, spleen, and ascites from mouse models, single-cell suspensions were prepared by tissue extraction, mashing, digestion with Liberase DL, TL (Roche, USA) and DNase І (Sigma-Aldrich, Germany). The erythrocytes were lysed with ACK lysis buffer (Gibco, Thermo Fisher Scientific), filtered by 40 μm pore size filter (Corning, NY, USA), incubated with 2% human Fc receptor blocking (eBioscience, Frankfurt, Germany), and stained with conjugated antibodies. Isotype IgG control was used in each experiment as reference. Flow cytometry was performed using BD Celesta, BD LSRII, or BD Fortessa (BD Bioscience, US). FACS was performed using BD Aria II or BD Melody (BD Bioscience, US). Data were analyzed using FlowJo software (Tree Star Inc., CA, USA).

### Human CD3^+^ T cell preparation

The human PBMCs were purchased from Stemcell Technologies (Cambridge, MA). CD3^+^ T cells were separated using CD3 microbeads (Miltenyi Biotec, USA) together with MACS columns and separators according to the manufacturer’s instructions. For stimulation of T cells, 24-well plates were coated with 10 μg/ml CD3 purified antibody. After washing with PBS twice, isolated CD3^+^ T cell were plated in CD3-coated plate in culture medium, and stimulated with CD28 purified antibody in the presence of 30 U/ml recombinant human IL-2 (Peprotech, USA).

### In vivo tumor models

Animal experimental protocols were in consistent with the Care and Use of Laboratory Animals Guide and approved by the Institutional Animal Care & Use Committee of Thomas Jefferson University (No. 01159). For establishing human ovarian cancer xenograft model in nude mice, 1 × 10^6^ A2780 cells suspended in 100 μl PBS with 50% Phenol Red-free Matrigel (Corning, NY, USA) were injected into flanks of 6-week-old female NCr nude mice (Taconic, US). Tail vein injections of Exo-con or Exo-NAC (100 μg in 100 μl PBS) were performed every 3 days. Tumors were measured with calipers and tumor volume was calculated with formula (width)^2^ × length/2. Mice were sacrificed 21 days after cell inoculation or the maximum tumor dimension reached 2.0 cm before 21 days.

For establishing immunocompetent mouse ovarian cancer model in C57BL/6 mice (Taconic), 8 × 10^6^ ID8-miR-con or ID8–155 mouse ovarian cancer tumor cells were inoculated intraperitoneally (i.p.) into C57/BL6 mice. Exosomes derived from ID8-miR-con (Exo-miR-con) or ID8-miR-155 (Exo-miR-155) tumor cells were prepared and injected via tail vein every 3 days. Starting at 60 days after tumor inoculation, mice were given IgG isotype control or anti-PDL1 antibodies at 200 μg/mouse every 3 days via intraperitoneal injection (IP). Mice were sacrificed when weight gain more than 30% and/or more than 40% waist circumference. Ascites, tumor nodules, spleens, and blood samples were collected and processed for further analysis.

### Statistical analysis

Data are shown as means ± SD. All experiments were repeated at least in triplicate. Statistical analysis were performed by Graph-Pad Prism software (Graph-Pad Software Inc., La Jolla, CA, USA). Differences between two groups were calculated using the unpaired two-tailed Student’s t-test. Statistical analyses of three or more groups were compared using one-way analysis of variance (ANOVA). Cumulative probabilities of overall survival were computed with the Kaplan−Meier analysis and comparisons between groups were analyzed using the log-rank test. *P* < 0.05 was considered statistically significant.

## Results

### ROS alter tumor exosomal miRNA signatures with miR-155-5p being the most downregulated miRNA

**Fig. 1:**
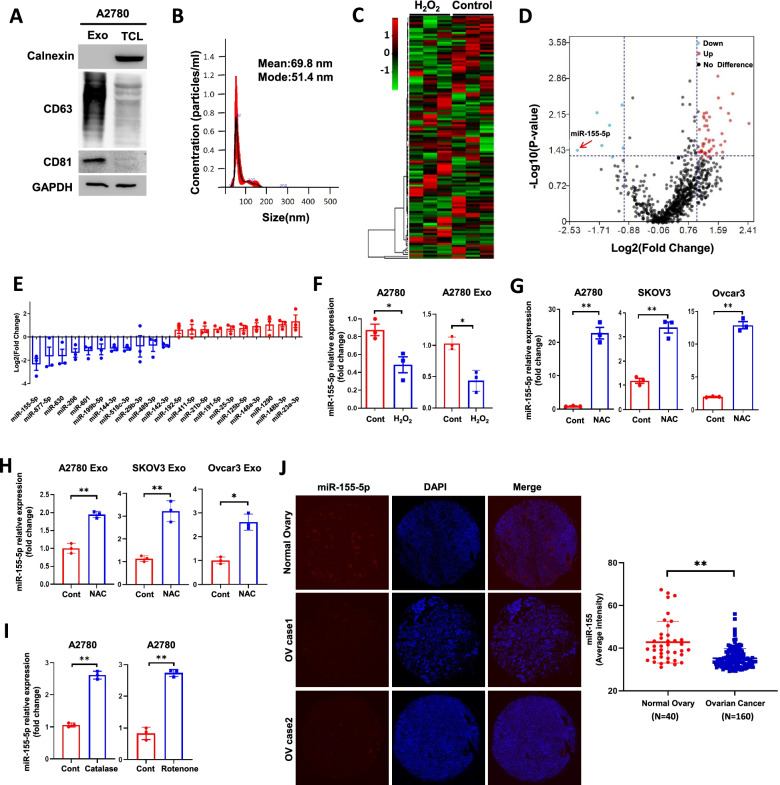
ROS alter exosomal miRNA signatures while miR-155-5p being the most downregulated miRNA. **A**. Exosomes were isolated from A2780 cells by ultracentrifugation. Exosomes (Exo) and total cell lysate (TCL) were analyzed by immunoblotting for CD63, CD81 and Calnexin. **B**. Exosomes isolated from A2780 cells were analyzed by nanoparticle tracking analysis (NTA). **C-E.** A2780 cells were treated with or without H_2_O_2_ at 100 μM for 4 h. 100 ng of exosomal RNAs were used as input for Nanostring nCounter miRNA analysis. Three biological replicates were used. The heatmap of exosome miRNAs profile (**C**), the volcano plot (**D**), and the fold changes of representative differentially expressed exosomal miRNAs in treated cells vs. control cells are shown (**E**). **F**. The expression levels of cellular and exosomal miR-155-5p were determined in A2780 cells treated with H_2_O_2_ (100 μM, 4 h). **G-H** The expression levels of cellular and exosomal miR-155-5p were determined in A2780, SKOV3, and Ovcar-3 cells treated with NAC (10 mM, 4 h). \**I**. The expression levels of cellular miR-155-5p were determined in A2780 cells treated with catalase-PEG (250 U/ml, 4 h) or rotenone (2.5 μM, 4 h). **J**. miR-155-5p expression was detected in human ovarian carcinoma tissue microarray (normal *N* = 40, ovarian cancer *N* = 160) by in situ hybridization using double DIG-labeled miRCURY LNA miRNA detection probes (red). Data are representative of 3 independent experiments. Data = Mean ± SD. *p < 0.05, **p < 0.01

We and others have shown that ROS alter the expression of miRNAs by regulating miRNA synthesis, transcription, and epigenetic modification [14, 15]. To determine whether ROS influence the TME through tumor exosomal miRNAs, we treated ovarian cancer A2780 cells with or without H_2_O_2,_ and purified A2780-derived exosomes characterized by positive exosomal markers CD63 and CD81, and a negative exosomal marker calnexin (Fig. 1A). The exosome size distribution was determined by nanoparticle tracking analysis (NTA) (Fig. 1B). The transmission electron microscope image revealed intact vesicles around 100 nm (right panel) in diameter (Fig. S1A). The exosomal miRNA profile determined by a NanoString nCounter microarray analysis, showed a number of differentially expressed miRNAs in H_2_O_2_-treated cells compared with those in control cells (Fig. 1C-E). Notably, exosomal miR-155-5p emerged as the most strongly down-regulated miRNA in H_2_O_2_-treated A2780 cells. We validated the microarray result by RT-qPCR by showing that H_2_O_2_ decreased both cellular miR-155-5p and exo-miR-155-5p expression levels (Fig. 1F). N-acetyl-L-cysteine (NAC) is a non-selective antioxidant which can similarly inhibit intracellular ROS as other scavengers such as catalase and rotenone. We observed ROS reductions in A2780 cells treated with NAC, catalase-PEG, and mitochondria complex I inhibitor rotenone with flow cytometry and fluorescent microscope using different dyes based on the ROS species (Fig.S1B, C). We then treated three ovarian cancer cell lines with NAC. As expected, both cellular miR-155-5p and exosomal miR-155-5p levels were decreased in ovarian cancer cells (Fig. 1G, H). To confirm that miR-155-5p is a bona fide ROS-sensitive miRNA, we also treated A2780 cells with catalase-PEG and rotenone. The expression levels of miR-155-5p were significantly increased with the ROS inhibitors (Fig. 1I).

miR-155-5p has been considered as an oncomiR in lymphoma, pancreatic, and oral squamous cell carcinoma cancers, and an oncosuppressor-miR in melanoma and colon cancer, as well as ovarian cancer [16–20]. To examine the abundance of miR-155-5p in ovarian cancer, we analyzed human ovarian carcinoma tissue microarray (TMA) for miR-155-5p expression by fluorescence in situ hybridization (FISH). Compared with normal ovarian tissues, the expression levels of miR-155-5p in ovarian cancer tissues were significantly lower (Fig. 1J), indicating a likely tumor suppressor role of miR-155-5p in ovarian cancer.

### NAC-derived tumor exosomes increase miR-155-5p levels in macrophages

**Fig. 2:**
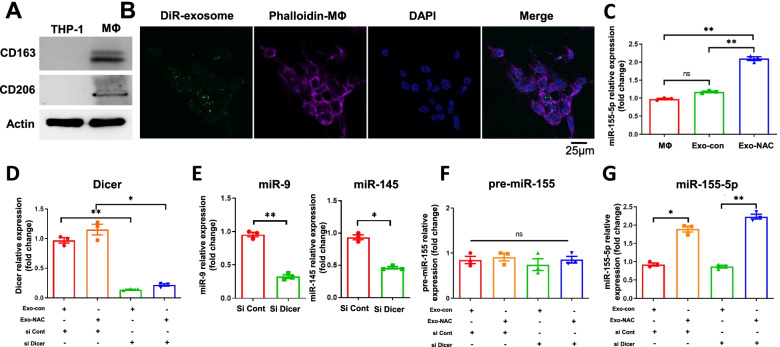
NAC-derived tumor exosomes increase miR-155-5p level in macrophages. **A**. THP-1 cells were treated with PMA at 100 nM for 24 h. The macrophage markers CD163 and CD206 were assessed by immunoblotting. **B**. Exosomes isolated from A2780 cells was labeled using lipophilic tracer DiR before being incubated with macrophages for 24 h. Cellular uptake of exosomes were examined by confocal microscopy. Macrophage nuclei were labeled with DAPI and actin cytoskeleton was labeled with Phalloidin. **C**. MΦ was treated with A2780-derived exosomes (Exo-con) and NAC-treated A2780-derived exosomes (Exo-NAC) at 100 μg/ml for 48 h. MiR-155-5p level was detected by RT-qPCR. **D-G**. Macrophages were transfected with DICER siRNASmartpool (si Dicer) or negative siRNA control (si Cont) for 24 h followed by Exo-con and Exo-NAC treatment for another 48 h. (**D**) Dicer mRNA expressions in macrophages were determined. (**E**) miR-145 and miR-9 expression levels were set as controls. (**F, G**) Pre-miR-155 and miR-155-5p levels were measured by qPCR in macrophages with treatments as indicated. Data are representative of three independent experiments. Data = Mean ± SD. *p < 0.05, **p < 0.01

TAMs are major types of stromal cells within the ovarian cancer TME. To determine whether tumor exosomes can be taken up by macrophages, we first allowed differentiation of THP-1 cells into CD206^+^ and CD163^+^ macrophages with phorbol 12-myristate 13-acetate (PMA) treatment (Fig. 2A). We then treated macrophages with DiR-labeled exosomes for 24 h. Exosomes were detectable in the cytosol of macrophages as visualized by confocal microscopy indicating these exosomes are taken in by macrophages (Fig. 2B). To determine the impact of tumor exosomes on macrophages, we treated macrophages (PMA-treated THP-1) with exosomes derived from NAC-treated A2780 cells (Exo-NAC) and exosomes derived from A2780 cells (Exo-con). The result showed elevated expression levels of miR-155-5p in Exo-NAC treated macrophages compared to Exo-con treated macrophages (Fig. 2C). Moreover, to rule out the possibility that de novo expression of miR-155-5p in macrophages is the source for the increased miR-155-5p, we knocked down Dicer in macrophages with siRNAs in order to disrupt the formation of mature miRNAs. As seen in Fig. 2D, the expression levels of Dicer were markedly reduced in the knockdown cells. Dicer knockdown greatly reduced expression of miRNAs, as evidenced by examples of miR-9 and miR-145 (Fig. 2E). Moreover, neither tumor exosomes nor Dicer knockdown affected pre-miR-155-5p expression levels (Fig. 2F). However, regardless of Dicer knockdown, levels of mature miR-155-5p increased with Exo-NAC treatment (Fig. 2G), demonstrating that the increased miR-155-5p in macrophages comes from tumor exosomes. Collectively, the results showed that inhibiting ROS in ovarian cancer cells increases the release of exo-miR-155-5p that is taken up by macrophages, resulting in the elevation of miR-155-5p.

### Exo-miR-155-5p inhibits macrophage migration and tumor infiltration in vitro

**Fig. 3:**
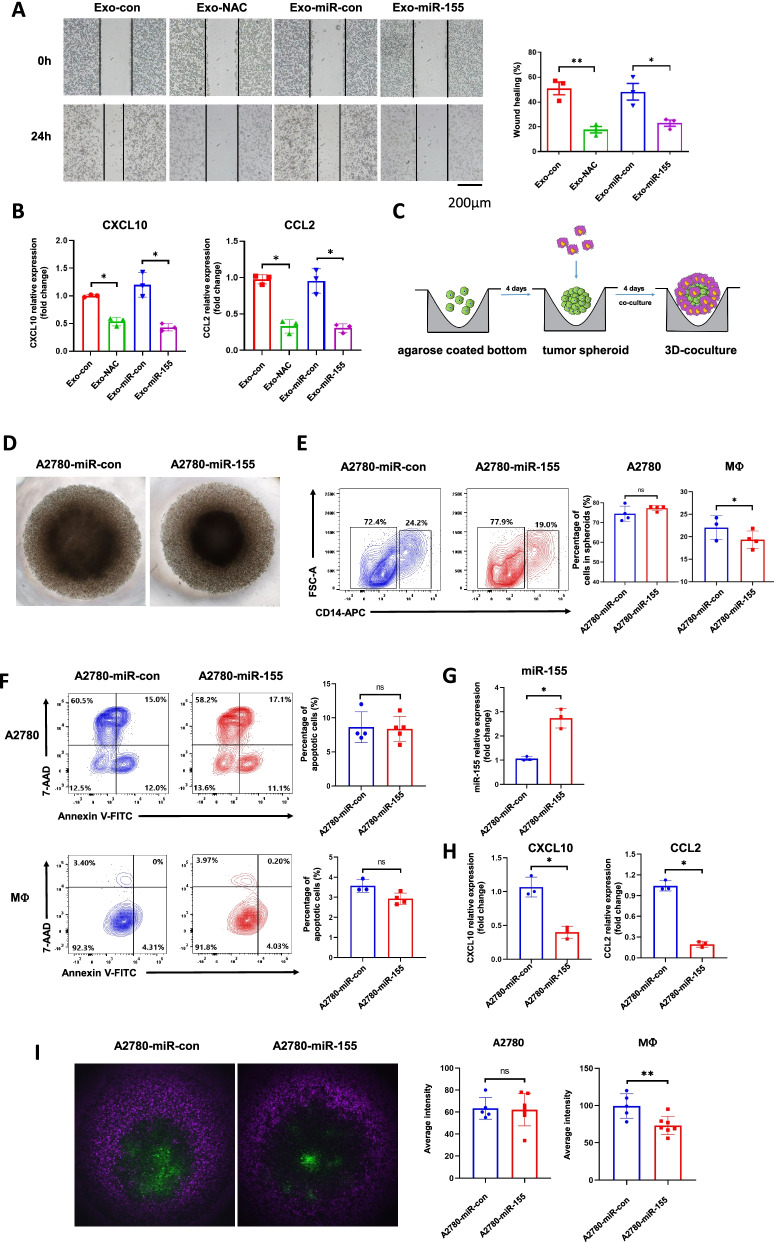
Exo-miR-155-5p inhibits macrophage migration and tumor infiltration. **A**. Macrophages were treated with Exo-con, Exo-NAC, Exo-miR-con (exosomes isolated from A2780-miR-con cells), or Exo-miR-155 (exosomes isolated from A2780-miR-155 stable cells), respectively for 48 h. The medium was then replaced and the cells were cultured for another 24 h after generating the scratches. Representative images are shown. **B**. Chemokines expressions in macrophages treated with Exo-con/Exo-NAC or Exo-miR-con/Exo-miR-155 were determined by RT-qPCR. **C**. The schematic diagram of the 3D co-culture model setup. A2780-miR-con and A2780-miR-155 stable cells were cultured to form tumor spheroid for 4 days. The same amount of macrophages were added and co-cultured for another 4 days. **D**. The representative images were shown after 4 days of 3D co-culture. **E**. The 3D co-cultures spheroids were re-suspended into single cell suspensions and analyzed by flow cytometry to determine the amounts of A2780 cells and infiltrated macrophages. **F**. The percentages of apoptotic A2780 cells and macrophages after co-culture were analyzed by flow cytometry. **G-H.** The CD14 positive infiltrated macrophages were isolated from 3D co-culture models using FACS. The expression levels of miR-155-5p (G) CXCL10, and CCL2 (H) were measured by RT-qPCR. **I**. A2780-miR-con and A2780-miR-155 stable cells were stained with cell tracker green before seeding onto 1% agarose concave surface for spheroid formation. Macrophages were stained by eFluor670 before adding into tumor spheroids. Representative images were taken by confocal microscope for 3D live cell imaging at 4 x magnification. The number of A2780 and infiltrated macrophages were analyzed as accumulative average fluorescent intensities. Green: A2780. Purple: macrophages. Data are representative of three independent experiments. Data = Mean ± SD. *p < 0.05, **p < 0.01

Next, we generated an A2780 cell line stably expressing miR-155-5p (A2780-miR-155) and miR-con cell line (A2780-miR-con). Expression levels of both cellular miR-155-5p and exosomal miR-155-5p were significantly higher in the A2780-miR-155 cells than in the control.

cells (Fig. S2A). To investigate if A2780-miR-155-derived exosomes (Exo-miR-155) affect macrophage migration, we performed a wound healing assay. The gap closure rates in macrophages treated with Exo-NAC or Exo-miR-155-5p for 24 h were markedly slower than their corresponding control cells. This indicates that Exo-NAC and Exo-miR-155-5p inhibit macrophage motility that is critical for macrophage recruitment into the TME (Fig. 3A). Furthermore, we examined a number of chemokines that are known to have chemotactic properties of various leukocyte subsets (Fig. S3A). We found CXCL10 and CCL2 were significantly decreased when treated with Exo-NAC or Exo-miR-155 in comparison to Exo-con or Exo-miR-con (Fig. 3B), suggesting these chemokines may contribute to macrophage mobilization.

A high degree of TAM infiltration in tumor tissues correlated with poor prognosis in many cancers. Next, we investigated whether tumor exo-miR-155-5p can reduce macrophage infiltration in vitro using a three-dimensional (3D) model to recapitulate the interaction between macrophages and ovarian cancer cells. First, we established 3D tumor spheroids using A2780-miR-con and A2780-miR-155 cells (Fig. 3C, S3B). A2780-miR-155 spheroids grew slower than A2780-miR-con as reflected by spheroid diameters 10 days after seeding, suggesting a tumor suppressive role of miR-155-5p. To build a co-culture model, A2780 cells were seeded onto non-adherent plates and were allowed to form spheroids for 4 days. Then, 20,000 CD14-positive THP-1 cells were added to the spheroids for an additional 4 days (Fig. 3D). Flow cytometry analysis showed the amounts of infiltrating macrophages in the A2780-miR-155 spheroids were significantly lower than those in the A2780-miR-con spheroids (Fig. 3E). To exclude the possibility that the decrease of macrophages was apoptotic-related, we conducted the apoptosis assay by Annexin V and 7-AAD staining, and found no significant change regarding the percentages of apoptotic tumor cells and macrophages (Fig. 3F). Then tumor cells and macrophages in the spheroids were sorted by FACS with the indicated gates followed by total RNA extraction (Fig. S3C). As expected, the miR-155-5p level increased in macrophages when co-cultured with A2780-miR-155 spheroids, indicating the transfer of exosomes from tumor cells (Fig. 3G). Then we analyzed the mRNA levels of chemokines associated with invasive phenotype in macrophages by RT-qPCR (Fig. 3H, S3D). Compared with macrophages in A2780-miR-con spheroids, the expression levels of CXCL10 and CCL2 significantly downregulated in macrophages in A2780-miR-155 spheroids (Fig. 3H). Among all the chemokines we detected in A2780 cells, CXCL10 and CCL2 levels were also lower in A2780-miR-155 cells sorted from spheroids (Fig. S3E). To observe the co-culture model in a spatiotemporal manner, we utilized cell tracker green-labeled A2780 spheroids and eFluor670-labeled infiltrating macrophages for 3D live cell imaging using confocal microscopy. In line with the results that we showed in Fig. 3E, fewer macrophages infiltrated into A2780-miR-155 spheroids compared with the control as reflected by lower fluorescent intensities (Fig. 3I, Movie. S1, S2). Taken together, these data suggest that ROS-related tumor exo-miR-155-5p is able to inhibit macrophage migration and infiltration.

### PD-L1 is a target of miR-155-5p in macrophages

**Fig. 4:**
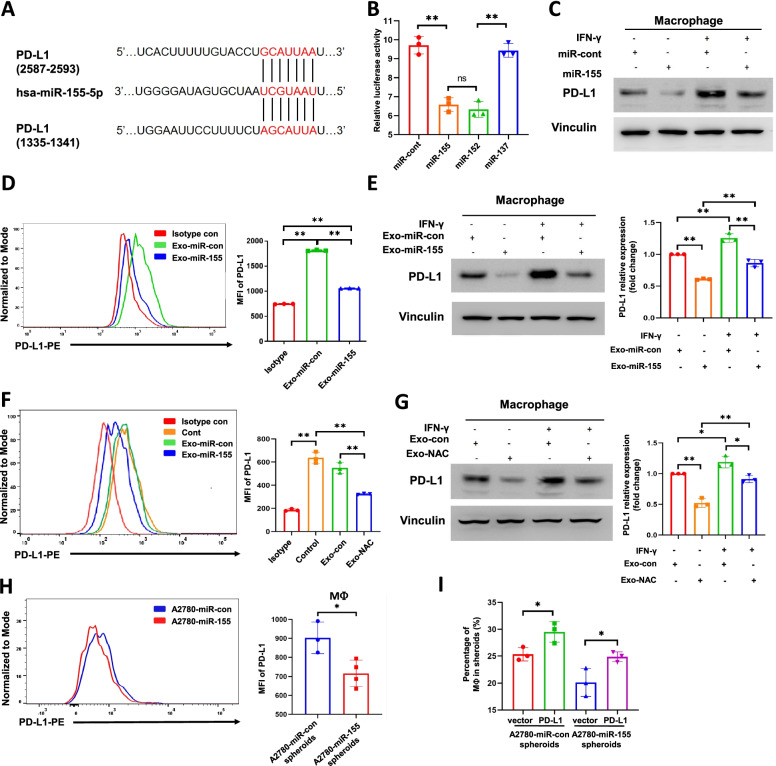
PD-L1 is a target of miR-155-5p in macrophages. **A**. Target Scan predicted 2 potential binding sites of miR-155-5p on PD-L1 3′-UTR. **B**. THP-1 cells were co-transfected with PD-L1 full-length 3’UTR plasmid and miR-cont, miR-155, miR-152 or miR-137. Luciferase activities were normalized by the Renilla control’s luciferase activity. miR-152 was set as a positive control, and miR-137 as a negative control. **C**. Macrophages were transfected with miRNA mimic control (miR-con) or miR-155-5p mimic (miR-155) for 48 h. PD-L1 expression was determined by immunoblotting. **D-E**. Macrophages pre-treated with IFN-γ (100 ng/ml, 24 h) were treated with Exo-miR-con or Exo-miR-155 at 100 μg/ml for 48 h. PD-L1 expression levels were determined by flow cytometry and immunoblotting. **F-G**. Macrophages pre-treated with IFN-γ (100 ng/ml, 24 h) were treated with Exo-con and Exo-NAC at 100 μg/ml for 48 h. PD-L1 expressions were detected by flow cytometry and immunoblotting. Mean Fluorescence Intensity (MFI) was statistically analyzed. **H**. PD-L1 expression levels in macrophages in A2780-miR-con spheroids and A2780-miR-155 spheroids as described in Fig. 4 were determined by flow cytometry. **I**. Macrophages were transiently transfected with PD-L1 or vector for 48 h before adding to the 3D tumor spheroids. The number of tumor infiltrating macrophages was determined by flow cytometry. Data are representative of 3 independent experiments. *p < 0.05, **p < 0.01

PD-L1 is a putative target of miR-155-5p with two potential miR-155-5p-binding sites in its 3′-UTR regions that are conserved in humans and mice (Fig. 4A). It was reported that miR-155-5p negatively regulates PD-L1 protein expression by directly binding the 3′ UTR of PD-L1 mRNA in human dermal lymphatic endothelial cells [21]. Interestingly, another study showed that miR-155 positively regulates the transcriptional activity of the PD-L1 gene in HEK-293 T cells [22]. To investigate the effect of miR-155-5p on PD-L1 expression levels, we co-transfected THP-1 cells with miR-cont, or miR-155-5p, and a PD-L1 3′ UTR full-length luciferase reporter. miR-152 served as positive control as it has been validated a direct target of PD-L1, andmiR-137 was used as a negative control since it does not contain predictive binding sites on PD-L1 3’UTR regions [23, 24]. Co-transfection of miR-155-5p or miR-152 with the PD-L1 reporter decreased luciferase activities while co-transfection of miR-cont or miR-137 did not affect luciferase activities, suggesting miR-155-5p inhibits PD-L1 expression at the transcriptional level in THP-1 cells (Fig. 4B). Moreover, overexpression of miR-155-5p decreased PD-L1 protein levels with or without IFN-ɣ induction (Fig. 4C).

To determine whether tumor exosomes inhibit PD-L1 protein levels in macrophages, we treated macrophages with Exo-miR-155 and Exo-miR-con, and found Exo-miR-155 treatment decreased PD-L1 expression significantly in macrophages compared to Exo-miR-con treatment (Fig. 4D). Immunoblotting showed that Exo-miR-155 decreased IFN-γ-induced PD-L1 protein levels in macrophages (Fig. 4E). We had shown that Exo-NAC treatment increased miR-155-5p in macrophages (Fig. 2D). In line with this result, Exo-NAC effectively suppressed PD-L1 protein levels in macrophages (Fig. 4F, G). In 3D coculture models, the PD-L1 expression levels in macrophages that were separated from A2780-miR-155 tumor spheroids were lower than those from A2780-miR-con spheroids (Fig. 4H). We transiently transfected a PD-L1 expressing plasmid or a control vector in macrophages for 48 h, then added into the tumor spheroids. We observed that overexpression of PD-L1 reversed the inhibition of miR-155-5p on macrophage tumor infiltration (Fig. 4I). These results suggest the uptake of tumor exo-155-5p reduced macrophage infiltration through PD-L1.

### NAC-derived tumor exosomes inhibit tumor growth and macrophage infiltration in nude mice

**Fig. 5:**
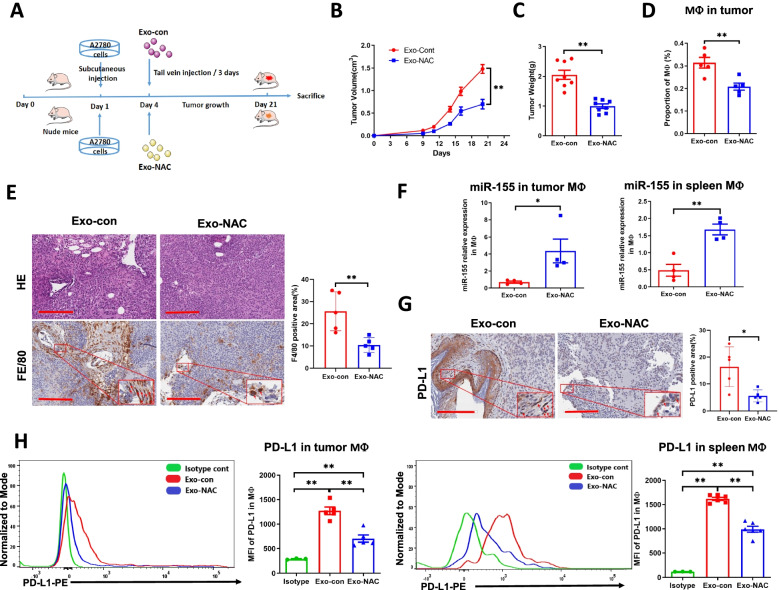
NAC-derived tumor exosomes reduce tumor growth and macrophage infiltration in nude mice. **A**. A2780 cells were injected into the flanks of 6-week-old female BALB/c nude mice. Tail vein injections of Exo-con or Exo-NAC (100 μg in 100 μl PBS) were performed every 3 days. Mice were terminated 21 days after the cell inoculation or the diameter of tumor reached 2.0 cm. **B-C**. Tumor growth curve and tumor weights are shown. *N* = 10 mice /group. **D**. The proportions of macrophages in tumors were measured by cell sorting (*n* = 5). **E**. Tumor tissues were sectioned and analyzed by IHC for macrophage marker F4/80 expression. Representative images of HE staining and IHC are shown (*n* = 5). Scale bar =200 μm. **F**. The miR-155-5p levels in tumor macrophages and splenic macrophages were measured by RT-qRCR (*n* = 4–5). **G**. Tumor tissues were sectioned and analyzed by IHC for PD-L1 expression. Representative images of IHC are shown (*n* = 5). Scale bar = 200 μm. **H**. Tumor macrophages and splenic macrophages were sorted and analyzed by flow cytometry for PD-L1 expression (*n* = 5). Isotype control: IgG. Data = Mean ± SD. *p < 0.05, **p < 0.01

Tumor xenografts were generated by A2780 cells by injection onto the flanks of female NCr nude mice. At 3 days after the implantation, Exo-con or Exo-NAC (100 μg in 100 μl PBS) was intravenously administered via the tail vein every 3 days, and tumor growth was monitored 3 times a week for 21 days (Fig. 5A). The mice treated with Exo-NAC grew smaller tumors compared to the mice treated with Exo-con (Fig. 5B, C). FACS was used to isolate macrophages from tumors (Fig. S4A) and spleens (Fig. S4B) in single cell suspensions with the indicated gates. The amount of tumor infiltrating macrophages in mice treated with Exo-NAC was significantly lower than in mice treated with Exo-con (Fig. 5D). The mouse macrophage marker F4/80 was also stained by IHC in tumor tissues (Fig. 5E), which showed similar results as illustrated in flow cytometry (Fig. 5D). The miR-155-5p expression level was significantly higher in macrophages isolated from tumors and spleens in mice treated with Exo-NAC (Fig. 5F), indicating miR-155-5p might be a major functional molecule in NAC-treated exosome cargo to downregulate PD-L1. As expected, the expression levels of PD-L1 in tumor tissues and tumor macrophages were significantly lower in mice treated with Exo-NAC (Fig. 5G, H). Collectively, these findings suggest that NAC-derived tumor exosomes are able to inhibit xenograft tumor growth and macrophage infiltration, in which the Exo-miR-155-5p/PD-L1 pathway might play a pivotal role.

### Macrophages treated with tumor exo-NAC or exo-miR-155-5p promote cytotoxic T cells activation through PD-L1 in vitro

**Fig. 6:**
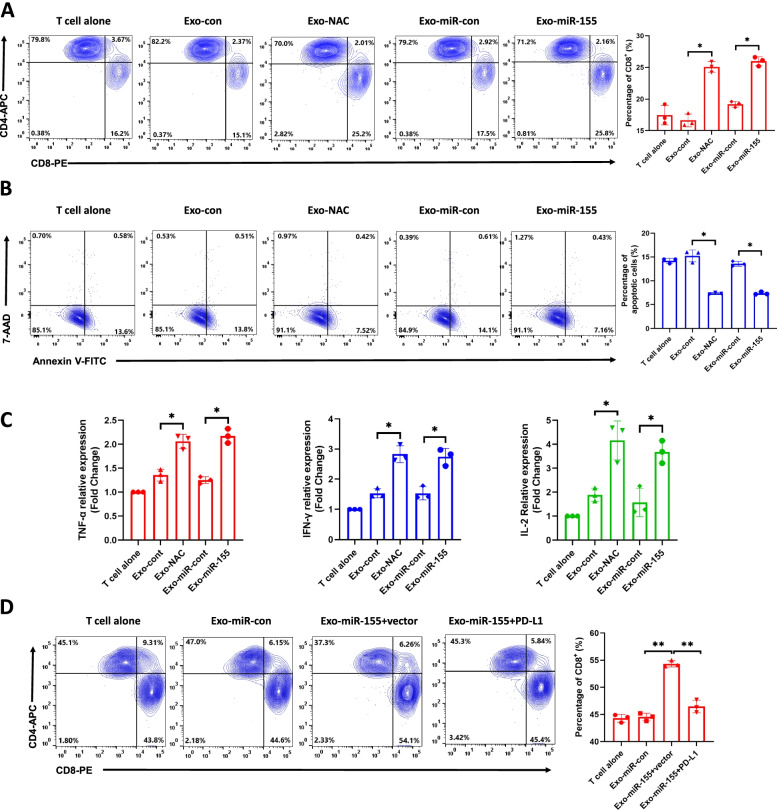
Exo-155-5p-treated macrophages activate cytotoxic T cells. **A-B**. Macrophages were pretreated with Exo-con, Exo-NAC, Exo-miR-con or Exo-miR-155 at 100 μg/ml for 48 h followed by co-cultured with activated T cells for another 48 h. Flow cytometry was performed to analyze CD8^+^ T cells proportion (A) and CD3^+^ T cell apoptosis (B). **C**. CD3^+^ T cells were sorted from the co-culture systems using FACS. Total RNA was extracted and the mRNA levels of chemokines were measured by RT-qPCR. **D**. Macrophages were transfected with PD-L1 or vector for 48 h before the Exo-miR-155 treatment followed by the co-culture with T cells for another 48 h. CD4^+^ and CD8^+^ T cells proportions were determined by flow cytometry. Data are representative of 3 independent experiments. Data = Mean ± SD. *p < 0.05, **p < 0.01. Abbreviations: Exo-con: Exo-con-treated macrophages + T cells; Exo-NAC: Exo-NAC-treated macrophages + T cells; Exo-miR-con: Exo-miR-con-treated macrophages + T cells; Exo-miR-155: Exo-miR-155-treated macrophages + T cells; Exo-miR-155 + PD-L1: macrophages overexpressed PD-L1 with Exo-miR-155 treatment + T cells

Accumulating evidence suggest that tumor-associated macrophage infiltration can promote tumor progression by impairing the immune responses of cytotoxic CD8^+^ T cells in the tumor microenvironment [25]. We asked whether macrophages treated with the exosomes impact T cell functions. To address this question, we isolated CD3^+^ T cells from human PMBC and activated T.

cells with CD3, CD28, and IL-2. Next, we co-cultured activated T cells with macrophages pre-treated with Exo-NAC, Exo-miR-155, or their respective controls for 24 h. The T cell function was evaluated by the percentage of cytotoxic CD8^+^ T cells (Fig. S5A) as well as the apoptosis of T cells using flow cytometry (Fig. S5B). Compared to the controls, Exo-NAC and Exo-miR-155 treated-macrophages significantly increased the CD8^+^ T cell proportion (Fig. 6A). The apoptosis rate of T cells after co-culture with exosome-treated macrophages was also examined by Annexin V/7-AAD staining. The results showed the percentage of apoptotic CD3^+^ T cells was significantly reduced after co-culturing with Exo-NAC and Exo-miR-155 treated macrophages compared with their respective controls (Fig. 6B).

Next, we isolated CD8^+^ T cells from the co-culture system to analyze the intracellular production of tumor necrosis factor alpha (TNF-α), gamma interferon (IFN-γ) and interleukin-2 (IL-2). The mRNA levels of these cytokines in CD8^+^ T cells were significantly increased after being co-cultured with Exo-NAC- and Exo-miR-155-treated macrophages (Fig. 6C). To determine whether CD3^+^ T cell activation is mediated by PD-L1, we transfected PD-L1 in macrophages before co-culturing with the T cells. We observed that PD-L1 overexpression effectively reversed an exo-miR-155-5p-mediated increase of CD8^+^ T cell (Fig. 6D), suggesting that Exo-miR-155-5p-treated macrophages can activate T cell functions by increasing CD8^+^ T cell ratio and decreasing T-cell apoptosis mainly through PD-L1 downregulation.

### Tumor exo-miR-155-5p inhibits tumor growth and macrophage infiltration and activates CD8 positive T cells in immune-competent mice

**Fig. 7:**
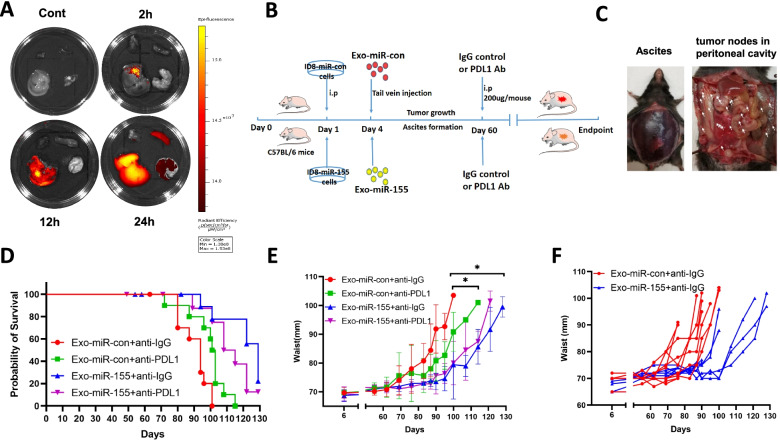
Exo-miR-155-5p suppresses tumor growth in immunocompetent mice. **A**. DiR-labeled exosomes (100 μg) were intravenously injected into C57/BL6 mice. The photos of liver, lung, spleen and heart were taken at 2, 12, and 24 h after the injection by In Vivo Imaging System (IVIS) spectrum. **B**. The schematic diagram of mice study design. **C**. Representative images of ascites and tumor nodes dissemination in peritoneal cavity. **D**. Kaplan-Meier survival curves among groups. Mice were terminated when they gained more than 40% waist circumference and/or 30% body weight or showed any signs of cachexia. Log rank P (Exo-miR-con+anti-IgG vs. Exo-miR-con+anti-PDL1) = 0.0132; P (Exo-miR-con+anti-IgG vs. Exo-miR-155 + anti- IgG) = 0.0002; P (Exo-miR-con+anti-PDL1 vs. Exo-miR-155 + anti-PDL1) = 0.0213; P (Exo-miR-155 + anti-IgG vs. Exo-miR-155 + anti-PDL1) = 0.1657. **E**. Waist circumference was measured weekly as an indicator of ascites accumulation (tumor progression). **F**. Individual waist measurements of mice over time (*n* = 9 in ID8-miR-con group, *n* = 10 in ID8-miR-155 group)

**Fig. 8:**
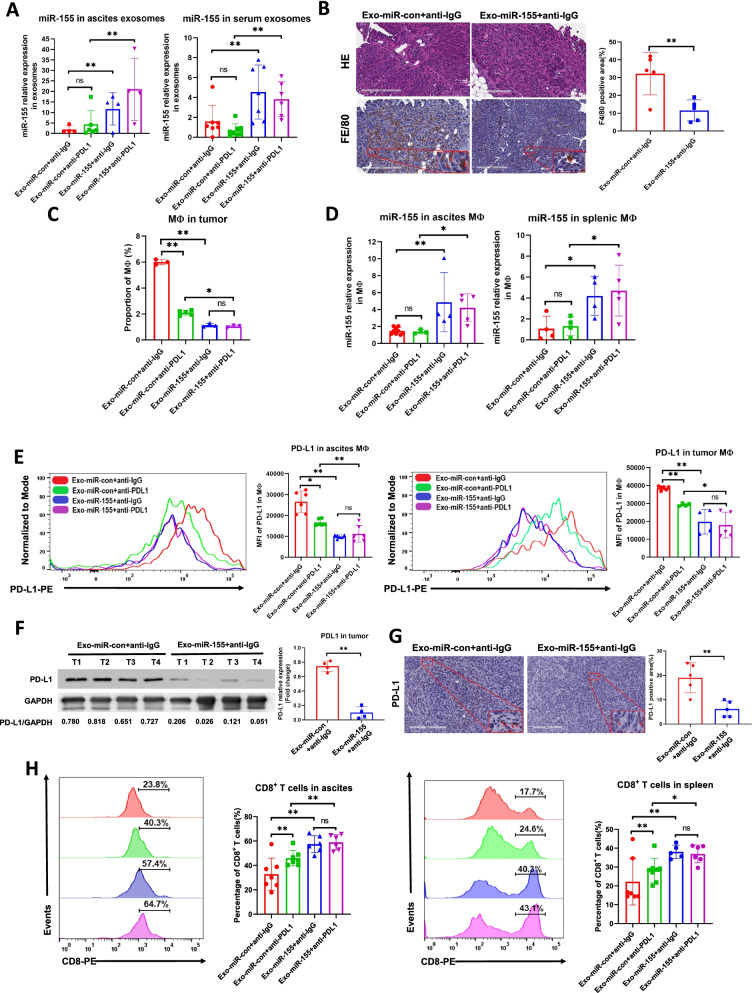
Exo-miR-155-5p activates CD8 positive T cells in vivo*.* **A**. Exosomes in ascites and serum were isolated and purified. The expression of exo-miR-155-5p was determined by RT-qPCR (*n* = 4–7). **B**. The expression of macrophage marker F4/80 was analyzed by IHC in tumor tissues. Representative images of HE staining and IHC are shown (*n* = 5). Scale bar = 200 μm. **C**. The proportions of macrophages in tumors were measured by cell sorting (*n* = 3–4). **D**. Macrophages were isolated from ascites and spleen using FACS followed by miR-155-5p detection with RT-qPCR (*n* = 3–6). **E**. Flow cytometry was performed to determine the PD-L1 expression in macrophages from ascites (*n* = 6), spleen (*n* = 4–5), and tumor tissues (*n* = 4–5). Mean Fluorescence Intensity (MFI) was statistically analyzed. **F**. The PD-L1 expressions were analyzed in tumor tissues by immunoblotting (*n* = 4/group). The densitometry of bands was quantified with image J. **G**. The expression PD-L1 in tumor tissue was determined by IHC. Representative images IHC are shown (*n* = 5). Scale bar = 200 μm. **H**. CD3^+^ CD8^+^ T cell subsets in ascites and spleen were analyzed by flow cytometry. The percentages of CD8^+^ cells were indicated (*n* = 6–7). Data = Mean ± SD. *p < 0.05, **p < 0.01

Next, we asked whether tumor exo-miR-155-5p could suppress tumor progression and activate the anti-tumor immune response in immune-competent mice. First, we examined the exosome distribution after in vivo delivery in C57BL/6 mice. The exosomes were detectable in the liver, spleen, and lung after 24 h (Fig. 7A). Next, we generated a murine ovarian cancer cell line ID8 stably expressing miR-155-5p (ID8-miR-155) and miR-con cell line (ID8-miR-con). The expression levels of both cellular miR-155-5p and exosomal miR-155-5p in ID8-miR-155 cells were 3-fold higher than those in control cells (Fig. S2B). We then established tumor models by intraperitoneal injection of ID8-miR-con or ID8-miR-155 cells into C57BL/6 mice. Exosomes from ID8-miR-con cells (Exo-miR-con) or exosomes from ID8-miR-155 cells (Exo-miR-155) were administered every 3 days, with anti-PD-L1 or anti-IgG antibodies administered starting at 60 days after tumor inoculation (Fig. 7B). Because ascites accumulation causes waist gain that can be used as an index of tumor progression in ID8 model [26], we monitored the tumor progression by measuring weight and waist circumference. Mice were euthanized when they gained more than 30% body weight and/or more than 40% waist circumference or showed any signs of cachexia or distress [26]. Mice in the control group developed visible ascites starting around 70 days after tumor implantation (Fig. 7C, left), and multiple tumor nodules were disseminated in the peritoneal cavity, including parietal and visceral surfaces (Fig. 7C, right). Delivery of Exo-miR-155 repressed tumor growth as evidenced by prolonged survival and delayed waist gain (Fig. 7D, E, F). Notably, Exo-miR-155 was more potent than anti-PD-L1 antibody treatment for inhibiting tumor growth. In addition, the combination of exo-miR-155-5p with anti-PD-L1 did not further improve the survival of the mice compared with exo-miR-155-5p treatment alone. These results indicated that additional factors are involved in miR-155-5p-mediated tumor suppression, and that the effects of anti-PD-L1 antibody and Exo-miR-155-5p on tumor growth may be repetitive instead of additive. Having demonstrated that tumor exo-miR-155-5p inhibited macrophage infiltration in vitro, we further investigated its effects on macrophage infiltration in vivo. Higher levels of miR-155-5p were found in exosomes isolated from ascites and serum in mice treated with Exo-miR-155, indicating a successful in vivo delivery of exo-miR-155-5p (Fig. 8A). The expression of macrophages marker F4/80 was lower in tumors in the Exo-miR-155-treated group by IHC (Fig. 8B). The proportion of macrophages in tumor samples was analyzed by FACS. The mice treated with either anti-PD-L1 antibody or exo-miR-155-5p had far fewer TAMs compared with the control mice (Fig. 8C). Moreover, the expression levels of miR-155-5p in ascites macrophages and spleen macrophages were elevated in the Exo-miR-155 group, suggesting tumor exo-miR-155-5p was uptaken by macrophages (Fig. 8D). Next, we examined PD-L1 expression in macrophages from ascites and tumors. Consistent with the findings obtained in nude mice, the expression of PD-L1 in ascites, and tumor macrophages were significantly lower in mice treated with Exo-miR-155 than in mice treated with Exo-miR-con (Fig. 8E). Similarly, the expression of PD-L1 in tumor tissue samples in the Exo-miR-155 group were generally downregulated determined by immunoblotting and IHC (Fig. 8F, G).

Lastly, we examined the effects of tumor exo-155-5p on T cell function in vivo. The percentages of CD8^+^ T cells from ascites and spleen in mice from Exo-miR-155 group were significantly increased compared with those in control groups (Fig. 8H). As expected, the anti-PD-L1 antibody increased the percentage of CD8^+^ T cells. Notably, adding anti-PD-L1 to Exo-miR-155 had no synergistic or additive effect on CD8^+^ T cells compared to Exo-miR-155 alone. Collectively, these in vivo findings indicate that exo-miR-155-5p inhibits tumor progression and prevent formation of the suppressive tumor microenvironment by reducing macrophage infiltration and subsequently activating T cell anti-tumor immune responses in ovarian cancer.

## Discussion

Here we provide evidence that the redox status of tumor cells serves as a critical regulator of the microenvironment in the control of immunologic mechanisms involved in their response to therapy. Compared with normal cells, cancer cells exhibit an increased level of intrinsic reactive oxygen stress because of mitochondrial dysfunction and metabolic alteration, reflecting a disorder of redox homeostasis [15, 27, 28]. Recent studies have shown that ROS promote tumor immune escape by creating an immunosuppressive tumor microenvironment [29, 30]. For example, excessive ROS production can reduce the infiltration of lymphocytes and facilitate recruitment and accumulation of regulatory T-cells and M2-like tumor-associated macrophages [30, 31]. A recent study showed that the inhibition of NADPH oxidase subunit NOX4 potentiates immunotherapy by preventing the formation of immunosuppressive phenotypes of cancer-associated fibroblast [32]. Consistent with this finding, we found inhibition of ROS by NAC was able to reprogram macrophages with tumor exosomes to suppress ovarian cancer development. We and other groups have shown that ROS-sensitive microRNAs contribute pivotally with respect to how cancer cells respond to ROS [14, 15, 33]. In this study, we identified exosomal miR-155-5p as a major downstream effector of ROS that mediates tumor immune responses. Given the dual roles of ROS in cancer development, the use of antioxidants in cancer treatment has had limited or even unexpected effects. A study showed that NAC and vitamin E accelerated lung cancer progression in a mouse model [34]. It is plausible that targeting downstream ROS-sensitive miRNAs, such as miR-155-5p, may provide a more specific approach to manipulation of ROS effects.

A key question in miRNA research is how their expression is regulated under specific contexts. Studies from different groups show that ROS are able to influence microRNAs through altering miRNA biogenesis, transcription factors, and epigenetic modulation [15]. Our prior study showed that endogenous ROS inhibit miR-199a and miR-125b genes expression through DNA hypermethylation in ovarian cancer cells [35]. However, the mechanisms by which ROS inhibit cellular and exosomal miR-155-5p levels in ovarian cancer cells are not clear. It is known that the transcription factor nuclear factor-κB (NF-κB), a redox-sensitive factor, can transactivate *miR-155-5p* gene expression [15]. Some studies have suggested that TNF-α-induced ROS accumulation decreases NF-κB expression and the level of its target miR-155-5p [36]. However, other groups argued that the rise of intracellular ROS levels, induced by TNF or IL-1, can up-regulate JNK-mediated NF-κB activation [37]. ROS production exerts opposing effects on NF-κB, inducing activation in the cytoplasm and inactivation in the nucleus [38]. To add complexity, reciprocal regulations exist between ROS and NF-κB signaling [39, 40]. Whether and how ROS regulate miR-155-5p through NF-κB remains unclear and is a topic worthy of further investigation.

TAMs represent the most abundant infiltrating immune cells in the peritoneal tumor microenvironment to influence ovarian cancer initiation, growth, and metastasis [41]. A meta-analysis showed that a high density of CD163^+^ TAMs infiltration was associated with poor prognosis in ovarian cancer [42]. It has been reported that exosomes derived from ovarian cancer cells are capable of educating macrophages to obtain the tumor-promoting M2 phenotype [43, 44]. We found tumor exosomal miR-155-5p inhibited tumor growth through macrophage infiltration inhibition and T cell activation. PD-L1 is an inhibitory checkpoint molecule known for its role in negative regulation of cytokine production and T cell function. PD-L1 is widely expressed in tumor cells, tumor infiltrating lymphocytes, and tumor stromal cells, especially tumor-associated CD68^+^ macrophages in ovarian cancer [45]. Expression of PD-L1 on dendritic cells and macrophages in ovarian cancer and melanoma patients correlated with the efficacy of treatment with either anti-PD-1 alone or in combination with anti-CTLA-4 [46]. However, it remains elusive how PD-L1 expression is upregulated in macrophages. Studies have shown that miR-155-5p inhibits PD-L1 expression by binding the 3′ UTR of PD-L1 mRNA [21, 22]. In this study, we found PD-L1 expression was lower in tumor and spleen macrophages in mice treated with Exo-miR-155-5p. Moreover, overexpression of PD-L1 reversed tumor exosomal miR-155-5p-induced CD8^+^ T cell proliferation, suggesting PD-L1 contributes to miR-155-5p-mediated anti-tumor immune response.

To date, the blockade of immune checkpoints in ovarian cancer has produced mixed results in preclinical setting and clinical trials [47, 48]. Krempski et al. reported that PD-1 antibody alone facilitated tumor regression and T cell function and activation while many others found a lack of response [49, 50]. In this study, we found PD-L1 antibody alone delayed tumor progression and ascites formation. Notably, exo-miR-155-5p inhibited tumor growth more potently than PD-L1 antibody, and the combination of the two agents failed to achieve better efficacy than exo-miR-155-5p alone. This indicates that PD-L1 inhibition by miR-155-5p may not entirely account for the immunosuppressive effects observed, suggesting that other pathways also contribute to miR-155-5p-mediated anti-tumor responses.

miR-155-5p has been recognized as a pro-inflammatory factor that can enhance the production of IL-1β, IL-6, IL-8, and TNF [51, 52]. It has been implicated in innate and adaptive anti-tumor immune response. Deletion of miR-155-5p reduces the capability of CD8^+^ cytotoxic T cells to respond to viral infection or tumor development [53], and aberrant expression of miR155-5p correlates with inflammation through targeting and degrading SHIP1 and WEE1 genes involved in inflammation [54, 55]. In addition to PD-L1, overexpression of miR-155-5p can reprogram tumor-associated macrophages to pro-inflammatory, antitumor macrophages, possibly by targeting C/EBP-ß and NF-ĸB and thus suppressing their signaling cascades [33, 56]. Our results showed that tumor exo-NAC or exo-miR-155-5p decreases CXCL10 and CCL2 expression levels in macrophages. Co-culture with macrophages treated with tumor exo-NAC or exo-miR-155-5p increased TNF-α, IFN-ɣ, and IL-2 levels in T cells. It remains to be investigated whether miR-155-5p regulate the chemokines directly or indirectly through PD-L1 or other pathways. Notably, the direct role of miR-155-5p in ovarian cancer cells is not clear yet. There is no significant change of cell proliferation in A2780-miRcont cells vs. A2780-miR-155-5p cells by MTT assay (72 h, data not shown). However, when co-cultured with macrophages in a 3D model, A2780-miR-155 cells in the spheroids grew slower than control cells starting at day 7 (Fig.S3B). In line with the mice study, we consider that miR-155-5p inhibits tumor growth mainly through the tumor microenvironment and adaptive immune response. However, this warrants further investigation.

## Conclusions

**Fig. 9:**
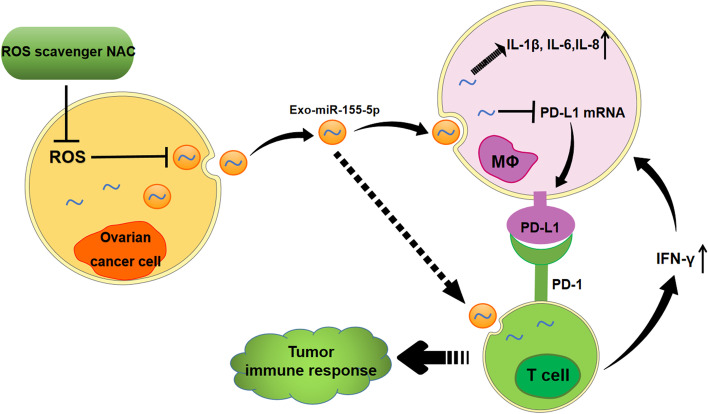
Graphic abstract. High level of ROS in cancer cells decrease tumor exosomal miR-155-5p that is taken up by macrophages to create an immunosuppressive microenvironment by upregulation of PD-L1 and other immunosuppressive factors that foster ovarian cancer development. Increase of tumor exo-miR-155 prevents ROS-mediated suppressive tumor immune response in ovarian cancer

We demonstrated a mechanism by which ovarian cancer cells inherently with high level of ROS decreases the amount of tumor exo-miR-155-5p that is taken up by macrophages to create an immunosuppressive microenvironment characterized by upregulation of PD-L1 and other immunosuppressive factors (Fig. 9). Understanding of the negative impact of ROS on the tumor immune response will improve current therapeutic strategies. Targeting miR-155-5p can be an alternative approach to prevent formation of ROS-mediated immunosuppressive TME. So far, the application of microRNAs in humans is hindered by lack of approaches to achieve high efficiency and safety concern for in vivo delivery of microRNA mimic or antimiR inhibitor. As exosomes may act as a promising and innovative nanocarrier for drug delivery characterized by their lower immunogenicity, but higher biocompatibility and greater circulation stability [57], the development of an exosome-mimicking liposome (EML) formulation of miR-155-5p that mimics the exosomes in which it is enclosed may have potential to provide significant clinical benefit to ovarian cancer patients.

## Supplementary Information

**Additional file 1.**

**Additional file 2.**

**Additional file 3.**

**Additional file 4.**

## Data Availability

The datasets used and/or analysed during the current study are available from the corresponding author on reasonable request.

## Abbreviations

Exo-miR-155-5p: Exosomes isolated from ovarian cancer cells stably expressing miR-155-5p
EOC: Epithelial ovarian cancer
IFN-γ: Gamma interferon
IL-2: Interleukin-2
NAC: N-acetylcysteine
NAC-treated exosomes (Exo-NAC): Exosomes isolated from ovarian cancer cells treated with NAC
ROS: Reactive oxygen species
TME: Tumor microenvironment
TAMs: Tumor-associated macrophages
TNF-α: Tumor necrosis factor alpha
PD-1: Programmed death protein 1
PD-L1: Programmed death-ligand 1

## References

[1] Siegel RL, Miller KD, Jemal A (2019). Cancer statistics, 2019. CA Cancer J Clin.

[2] Coburn SB, Bray F, Sherman ME, Trabert B (2017). International patterns and trends in ovarian cancer incidence, overall and by histologic subtype. Int J Cancer.

[3] Tewari KS, Java JJ, Eskander RN, Monk BJ, Burger RA (2016). Early initiation of chemotherapy following complete resection of advanced ovarian cancer associated with improved survival: NRG oncology/gynecologic oncology group study. Ann Oncol.

[4] Paclitaxel plus carboplatin versus standard chemotherapy with either single-agent carboplatin or cyclophosphamide, doxorubicin, and cisplatin in women with ovarian cancer: the ICON3 randomised trial. Lancet (London, England). 2002;360(9332):505–15. 10.1016/S0140-6736(02)09738-6 12241653

[5] Ahmed N, Stenvers KL (2013). Getting to know ovarian cancer ascites: opportunities for targeted therapy-based translational research. Front Oncol.

[6] Takaishi K, Komohara Y, Tashiro H, Ohtake H, Nakagawa T, Katabuchi H, Takeya M (2010). Involvement of M2-polarized macrophages in the ascites from advanced epithelial ovarian carcinoma in tumor progression via Stat3 activation. Cancer Sci.

[7] Yousefzadeh Y, Hallaj S, Baghi Moornani M, Asghary A, Azizi G, Hojjat-Farsangi M, Ghalamfarsa G, Jadidi-Niaragh F (2020). Tumor associated macrophages in the molecular pathogenesis of ovarian cancer. Int Immunopharmacol.

[8] Roma-Rodrigues C, Fernandes AR, Baptista PV (2014). Exosome in tumour microenvironment: overview of the crosstalk between normal and cancer cells. Biomed Res Int.

[9] Kosaka N, Yoshioka Y, Fujita Y, Ochiya T (2016). Versatile roles of extracellular vesicles in cancer. J Clin Invest.

[10] Sun Z, Shi K, Yang S, Liu J, Zhou Q, Wang G, Song J, Li Z, Zhang Z, Yuan W (2018). Effect of exosomal miRNA on cancer biology and clinical applications. Mol Cancer.

[11] Whiteside TL, Makowski GS (2016). Chapter Four - Tumor-Derived Exosomes and Their Role in Cancer Progression. Advances in Clinical Chemistry.

[12] Eldh M, Ekström K, Valadi H, Sjöstrand M, Olsson B, Jernås M, Lötvall J (2010). Exosomes communicate protective messages during oxidative stress; possible role of exosomal shuttle RNA. PLoS One.

[13] Xia C, Meng Q, Liu LZ, Rojanasakul Y, Wang XR, Jiang BH (2007). Reactive oxygen species regulate angiogenesis and tumor growth through vascular endothelial growth factor. Cancer Res.

[14] He J, Jiang BH (2016). Interplay between reactive oxygen species and MicroRNAs in Cancer. Curr Pharmacol Rep.

[15] Lu C, Zhou D, Wang Q, Liu W, Yu F, Wu F, Chen C (2020). Crosstalk of MicroRNAs and oxidative stress in the pathogenesis of Cancer. Oxidative Med Cell Longev.

[16] Liu J, Chen Z, Xiang J, Gu X (2018). MicroRNA-155 acts as a tumor suppressor in colorectal cancer by targeting CTHRC1 in vitro. Oncol Lett.

[17] Martinez-Usatorre A, Sempere LF, Carmona SJ, Carretero-Iglesia L, Monnot G, Speiser DE, Rufer N, Donda A, Zehn D, Jandus C (2019). MicroRNA-155 expression is enhanced by T-cell receptor stimulation strength and correlates with improved tumor control in melanoma. Cancer Immunol Res.

[18] Tili E, Croce CM, Michaille J-J (2009). miR-155: on the crosstalk between inflammation and Cancer. Int Rev Immunol.

[19] Yadav S, Singh N, Shah PP, Rowbotham DA, Malik D, Srivastav A, Shankar J, Lam WL, Lockwood WW, Beverly LJ (2017). MIR155 Regulation of Ubiquilin1 and Ubiquilin2: Implications in Cellular Protection and Tumorigenesis. Neoplasia (New York, NY).

[20] Chen W, Huang L, Hao C, Zeng W, Luo X, Li X, Zhou L, Jiang S, Chen Z, He Y (2016). MicroRNA-155 promotes apoptosis in SKOV3, A2780, and primary cultured ovarian cancer cells. Tumour Biol.

[21] Yee D, Shah KM, Coles MC, Sharp TV, Lagos D (2017). MicroRNA-155 induction via TNF-alpha and IFN-gamma suppresses expression of programmed death ligand-1 (PD-L1) in human primary cells. J Biol Chem.

[22] Zheng Z, Sun R, Zhao HJ, Fu D, Zhong HJ, Weng XQ, Qu B, Zhao Y, Wang L, Zhao WL (2019). MiR155 sensitized B-lymphoma cells to anti-PD-L1 antibody via PD-1/PD-L1-mediated lymphoma cell interaction with CD8+T cells. Mol Cancer.

[23] Wang Q, Lin W, Tang X, Li S, Guo L, Lin Y, Kwok HF (2017). The roles of microRNAs in regulating the expression of PD-1/PD-L1 immune checkpoint. Int J Mol Sci.

[24] Wang Y, Wang D, Xie G, Yin Y, Zhao E, Tao K, Li R (2017). MicroRNA-152 regulates immune response via targeting B7-H1 in gastric carcinoma. Oncotarget.

[25] Qian BZ, Pollard JW (2010). Macrophage diversity enhances tumor progression and metastasis. Cell.

[26] Czystowska-Kuzmicz M, Sosnowska A, Nowis D, Ramji K, Szajnik M, Chlebowska-Tuz J, Wolinska E, Gaj P, Grazul M, Pilch Z (2019). Small extracellular vesicles containing arginase-1 suppress T-cell responses and promote tumor growth in ovarian carcinoma. Nat Commun.

[27] Leisegang MS, Schröder K, Brandes RP (2018). Redox regulation and noncoding RNAs. Antioxid Redox Signal.

[28] Tong L, Chuang CC, Wu S, Zuo L (2015). Reactive oxygen species in redox cancer therapy. Cancer Lett.

[29] Lin X, Zheng W, Liu J, Zhang Y, Qin H, Wu H, Xue B, Lu Y, Shen P (2013). Oxidative stress in malignant melanoma enhances tumor necrosis factor-α secretion of tumor-associated macrophages that promote cancer cell invasion. Antioxid Redox Signal.

[30] Weinberg F, Ramnath N, Nagrath D. Reactive oxygen species in the tumor microenvironment: an overview. Cancers (Basel). 2019;11(8). 10.3390/cancers11081191 PMC6721577 31426364

[31] Kraaij MD, Savage ND, van der Kooij SW, Koekkoek K, Wang J, van den Berg JM, Ottenhoff TH, Kuijpers TW, Holmdahl R, van Kooten C (2010). Induction of regulatory T cells by macrophages is dependent on production of reactive oxygen species. Proc Natl Acad Sci U S A.

[32] Ford K, Hanley CJ, Mellone M, Szyndralewiez C, Heitz F, Wiesel P, Wood O, Machado M, Lopez MA, Ganesan AP (2020). NOX4 inhibition potentiates immunotherapy by overcoming Cancer-associated fibroblast-mediated CD8 T-cell exclusion from tumors. Cancer Res.

[33] Wang M, Yang F, Qiu R, Zhu M, Zhang H, Xu W, Shen B, Zhu W (2018). The role of mmu-miR-155-5p-NF-κB signaling in the education of bone marrow-derived mesenchymal stem cells by gastric cancer cells. Cancer Med.

[34] Sayin VI, Ibrahim MX, Larsson E, Nilsson JA, Lindahl P, Bergo MO (2014). Antioxidants accelerate lung cancer progression in mice. Sci Transl Med.

[35] He J, Xu Q, Jing Y, Agani F, Qian X, Carpenter R, Li Q, Wang XR, Peiper SS, Lu Z (2012). Reactive oxygen species regulate ERBB2 and ERBB3 expression via miR-199a/125b and DNA methylation. EMBO Rep.

[36] Sakon S, Xue X, Takekawa M, Sasazuki T, Okazaki T, Kojima Y, Piao JH, Yagita H, Okumura K, Doi T (2003). NF-kappaB inhibits TNF-induced accumulation of ROS that mediate prolonged MAPK activation and necrotic cell death. EMBO J.

[37] Lo YY, Wong JM, Cruz TF (1996). Reactive oxygen species mediate cytokine activation of c-Jun NH2-terminal kinases. J Biol Chem.

[38] Kabe Y, Ando K, Hirao S, Yoshida M, Handa H (2005). Redox regulation of NF-kappaB activation: distinct redox regulation between the cytoplasm and the nucleus. Antioxid Redox Signal.

[39] Morgan MJ, Liu Z-G (2011). Crosstalk of reactive oxygen species and NF-κB signaling. Cell Res.

[40] Hoesel B, Schmid JA (2013). The complexity of NF-κB signaling in inflammation and cancer. Mol Cancer.

[41] Colvin EK (2014). Tumor-associated macrophages contribute to tumor progression in ovarian cancer. Front Oncol.

[42] Yuan X, Zhang J, Li D, Mao Y, Mo F, Du W, Ma X (2017). Prognostic significance of tumor-associated macrophages in ovarian cancer: a meta-analysis. Gynecol Oncol.

[43] Ying X, Wu Q, Wu X, Zhu Q, Wang X, Jiang L, Chen X, Wang X (2016). Epithelial ovarian cancer-secreted exosomal miR-222-3p induces polarization of tumor-associated macrophages. Oncotarget.

[44] Chen X, Ying X, Wang X, Wu X, Zhu Q, Wang X (2017). Exosomes derived from hypoxic epithelial ovarian cancer deliver microRNA-940 to induce macrophage M2 polarization. Oncol Rep.

[45] Liu J, Fan L, Yu H, Zhang J, He Y, Feng D, Wang F, Li X, Liu Q, Li Y (2019). Endoplasmic reticulum stress causes liver Cancer cells to release Exosomal miR-23a-3p and up-regulate programmed death ligand 1 expression in macrophages. Hepatology.

[46] Lin H, Wei S, Hurt EM, Green MD, Zhao L, Vatan L, Szeliga W, Herbst R, Harms PW, Fecher LA (2018). Host expression of PD-L1 determines efficacy of PD-L1 pathway blockade-mediated tumor regression. J Clin Invest.

[47] Doo DW, Norian LA, Arend RC (2019). Checkpoint inhibitors in ovarian cancer: a review of preclinical data. Gynecol Oncol Rep.

[48] Zhu X, Lang J (2016). The significance and therapeutic potential of PD-1 and its ligands in ovarian cancer: a systematic review. Gynecol Oncol.

[49] Krempski J, Karyampudi L, Behrens MD, Erskine CL, Hartmann L, Dong H, Goode EL, Kalli KR, Knutson KL (2011). Tumor-infiltrating programmed death receptor-1+ dendritic cells mediate immune suppression in ovarian cancer. J Immunol (Baltimore, Md : 1950).

[50] Wei H, Zhao L, Li W, Fan K, Qian W, Hou S, Wang H, Dai M, Hellstrom I, Hellstrom KE (2013). Combinatorial PD-1 blockade and CD137 activation has therapeutic efficacy in murine Cancer models and synergizes with cisplatin. PLoS One.

[51] Jin HM, Kim TJ, Choi JH, Kim MJ, Cho YN, Nam KI, Kee SJ, Moon JB, Choi SY, Park DJ (2014). MicroRNA-155 as a proinflammatory regulator via SHIP-1 down-regulation in acute gouty arthritis. Arthritis Res Therapy.

[52] Kurowska-Stolarska M, Alivernini S, Ballantine LE, Asquith DL, Millar NL, Gilchrist DS, Reilly J, Ierna M, Fraser AR, Stolarski B (2011). MicroRNA-155 as a proinflammatory regulator in clinical and experimental arthritis. Proc Natl Acad Sci U S A.

[53] Dudda JC, Salaun B, Ji Y, Palmer DC, Monnot GC, Merck E, Boudousquie C, Utzschneider DT, Escobar TM, Perret R (2013). MicroRNA-155 is required for effector CD8+ T cell responses to virus infection and cancer. Immunity.

[54] Tili E, Michaille JJ, Wernicke D, Alder H, Costinean S, Volinia S, Croce CM (2011). Mutator activity induced by microRNA-155 (miR-155) links inflammation and cancer. Proc Natl Acad Sci U S A.

[55] O'Connell RM, Chaudhuri AA, Rao DS, Baltimore D (2009). Inositol phosphatase SHIP1 is a primary target of miR-155. Proc Natl Acad Sci U S A.

[56] Michaille JJ, Awad H, Fortman EC, Efanov AA, Tili E (2019). miR-155 expression in antitumor immunity: the higher the better?. Genes Chromosom Cancer.

[57] Lu M, Zhao X, Xing H, Xun Z, Zhu S, Lang L, Yang T, Cai C, Wang D, Ding P (2018). Comparison of exosome-mimicking liposomes with conventional liposomes for intracellular delivery of siRNA. Int J Pharm.

