# Supplementary material for: Reactive oxygen species reprogram macrophages to suppress antitumor immune response through the exosomal miR-155-5p/PD-L1 pathway
Source: J Exp Clin Cancer Res. 2022 Jan 27;41:41. doi: 10.1186/s13046-022-02244-1 (PMC8793215; doi:10.1186/s13046-022-02244-1)
Supplement: Supplementary file 1 — Additional file 1. [file 13046_2022_2244_MOESM1_ESM.docx]

**Supplementary figures**

**
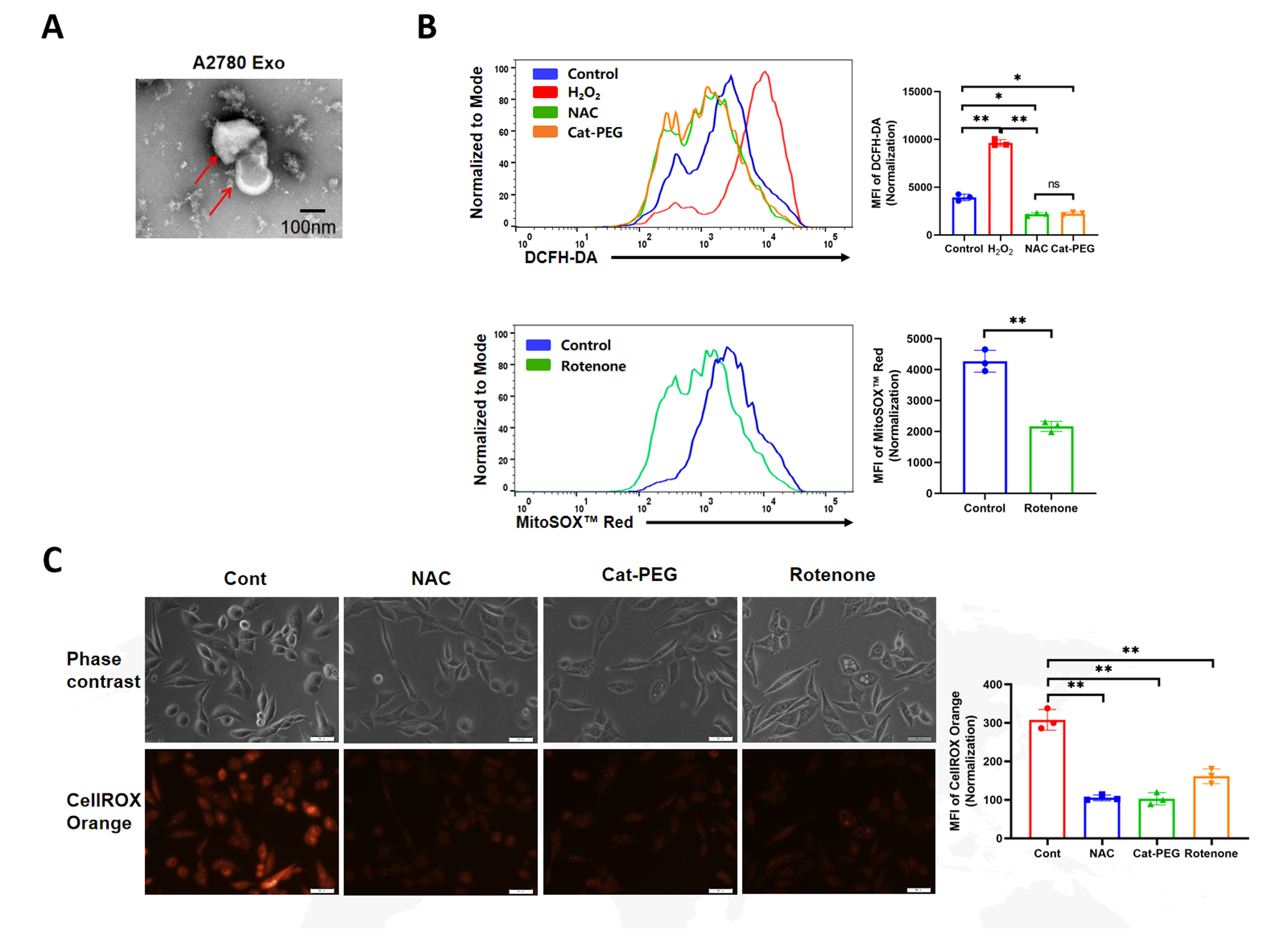
**

**Fig.S1 ROS detection in cells treated with the ROS inhibitors.**

1. A representative transmission electron microscopes (TEM) image of purified A2780-derived exosomes.

**B.** ROS levels in A2780 cells treated with H_2_O_2_ (100 μM)_,_ NAC (10 mM), catalase-PEG (250 U/ml), and rotenone (2.5 µM) for 4 hours were detected by flow cytometry using DCFH-DA or MitoSOX^TM^ Red, respectively.

**C.** ROS levels in A2780 cells treated with NAC, catalase-PEG, and rotenone were detected by fluorescent microscope using CellROX Orange. The fluorescent intensity was quantified using ImageJ software. Data are representative of 3 independent experiments. *p<0.05, **p<0.01. Scale bar = 50 µm.

**
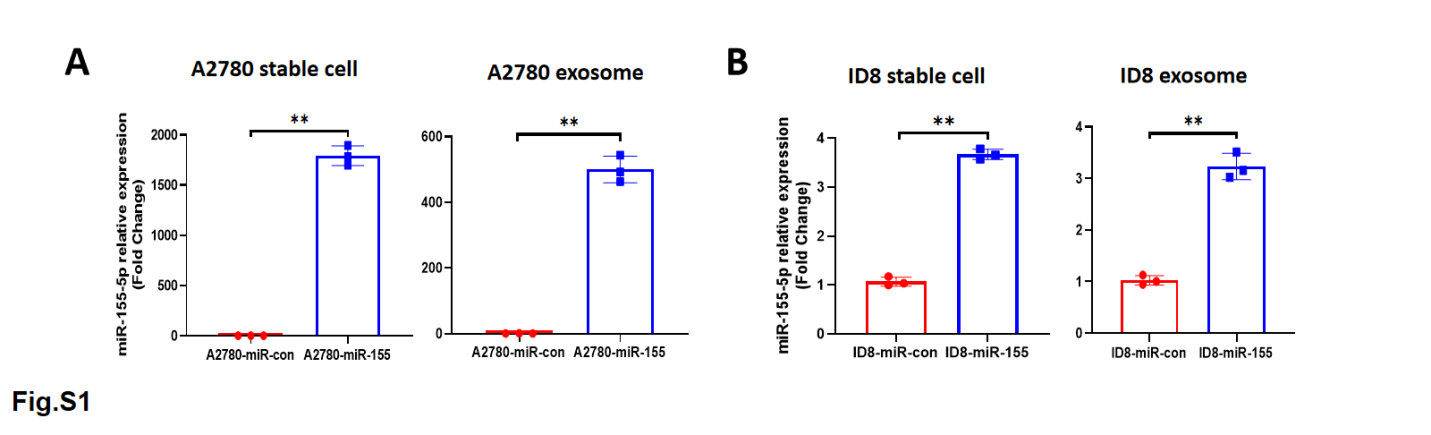
**

**Fig.S2 Generation of A2780 cells stably expressing miR-155-5p.**

**A**. A2780 cells were transfected with a miR-155-5p expression plasmid followed by G418 selection. The expression levels of cellular and exosomal miR-155-5p were determined by RT-qPCR.

**B**. ID8 mouse ovarian cancer cells were transfected with a miR-155-5p expression plasmid followed by G418 selection. The expression levels of cellular and exosomal miR-155-5p were determined by RT-qPCR. Data are representative of 3 independent experiments. *p<0.05, **p<0.01.


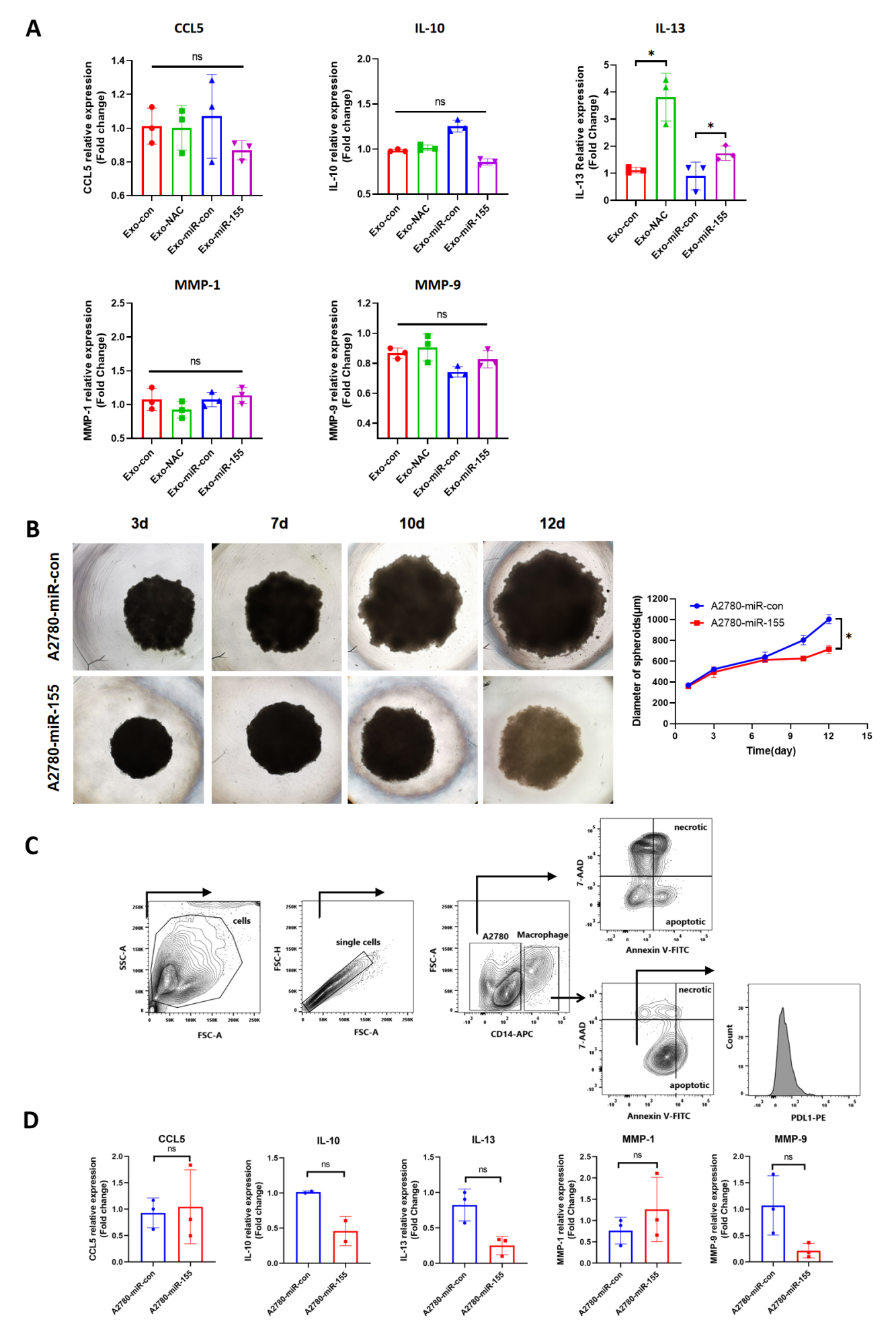


**
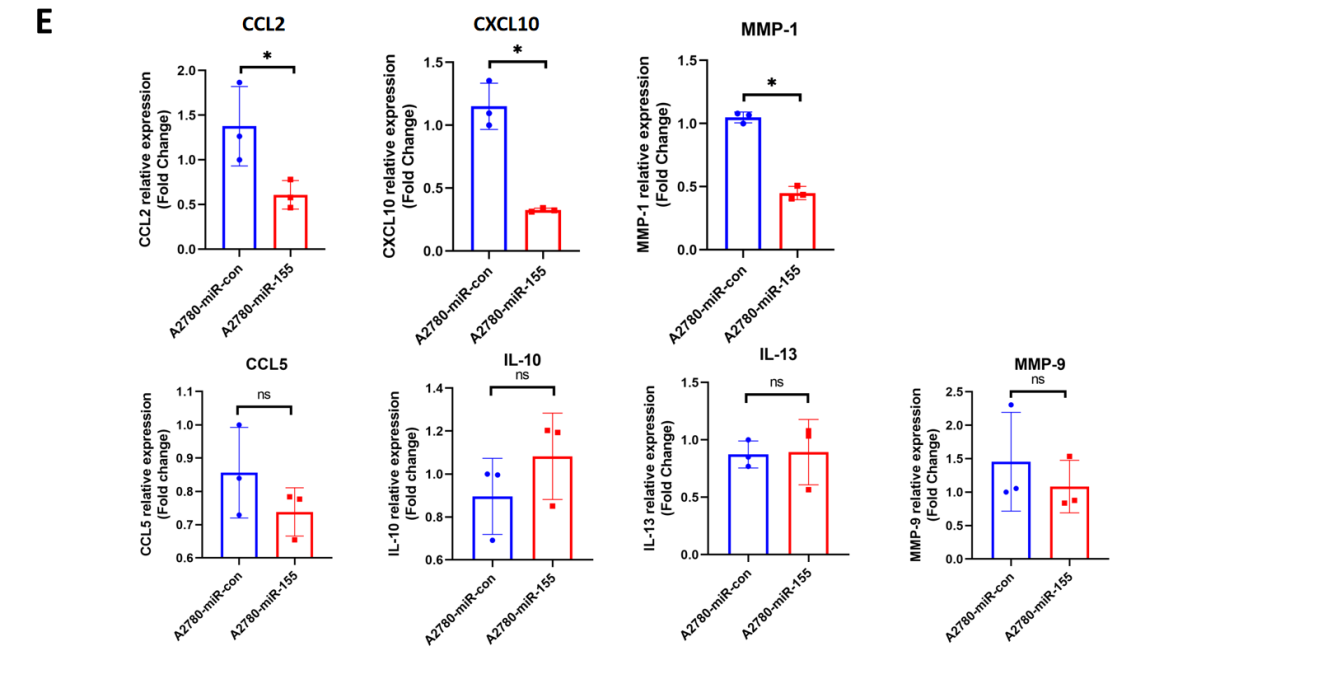
**

**Fig.S3 Co-culture of A2780 tumor spheroids and macrophages**

**A**. Chemokines expressions in macrophages in migration assay treated with Exo-cont / Exo-NAC or Exo-miR-con / Exo-miR-155 were determined by RT-qPCR. Data are representative of 3 independent experiments. *p<0.05, **p<0.01.

**B**. Tumor spheroids were generated by A2780-miR-con and A2780-miR-155 cells. The diameter of spheroids was measured at 3d, 7d, 10d and 12d, respectively.

**C-D.** 1×10^5^ macrophages were added per well after tumor spheroids formation. The percentages of apoptotic cells were analyzed by 7-AAD and Annexin V staining. The macrophages were further sorted for PD-L1 expression (C). The CD14 positive infiltrated macrophages were isolated from 3D co-culture model using fluorescence-activated cell sorting (FACS). The expression levels of CCL5, IL-10, IL-13, MMP-1, MMP-9 were measured by RT-qPCR (D).

**E**. A2780 cells were isolated from single cell suspension of the formed spheroids and infiltrated macrophages by FACS. RT-qPCR was performed to measure the mRNA level of chemokines in A2780 cells. Data are representative of 3 independent experiments. *p<0.05, **p<0.01.


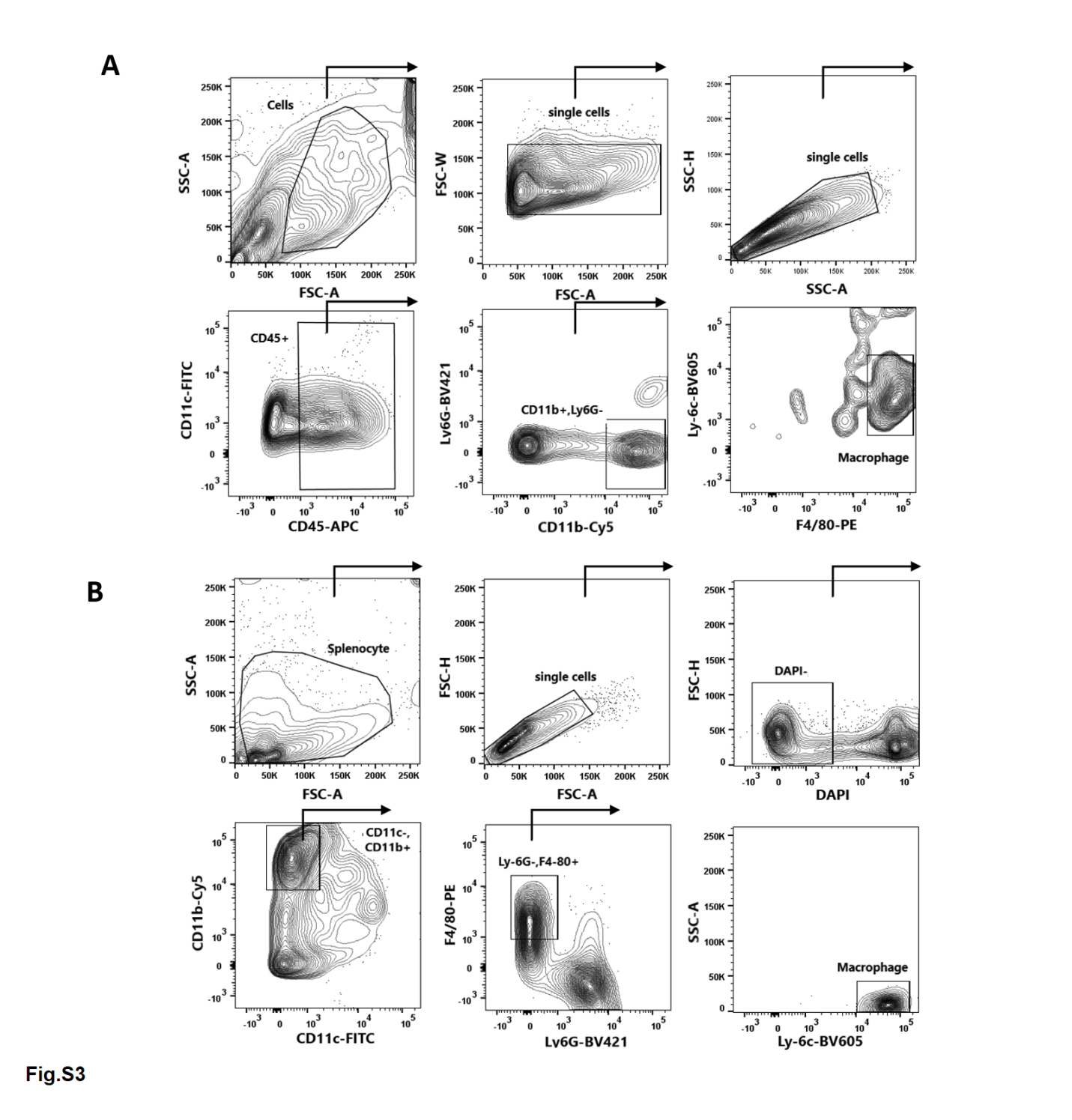


# Fig.S4 Gating strategy for tumor infiltrated macrophages and spleen macrophages via flow cytometry.

**A**. Single cell suspensions from tumors were first gated using FSC and SSC, and then gated for CD45^+^ immune cells. Macrophages and monocytes were defined as CD11b^+^Ly6G^-^ cells, which were further gated on F4/80 high macrophages.

**B**. After defining live cells from spleen as DAPI^-^, CD11b^+^CD11c high dendritic cells were then excluded followed by the exclusion of Ly6G^+^ neutrophils. Macrophages were further gated as F4/80 high Ly6C^+^.


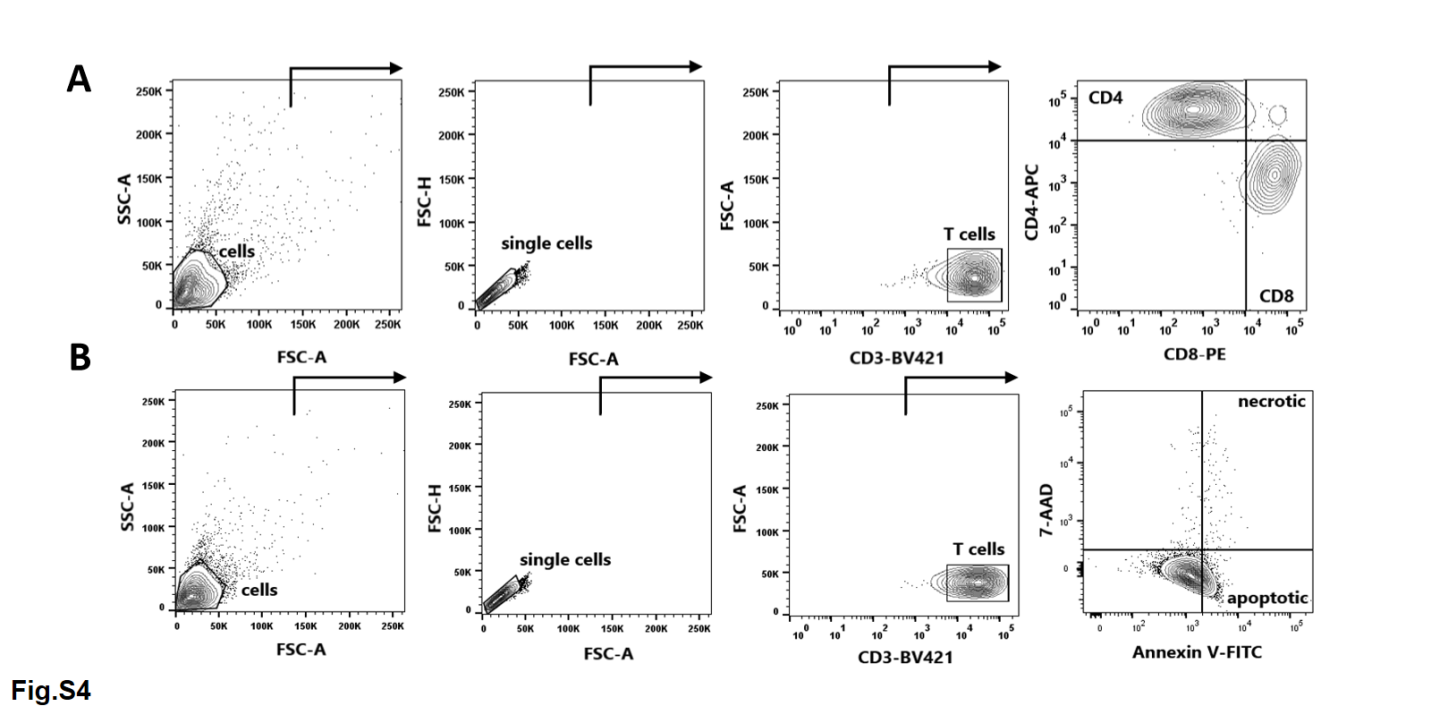


# Fig.S5 Flow cytometric gating strategy for detection of percentage and apoptosis of T cells

**A**. The lymphocytes were determined by the forward and side scatter profile, and the CD3^+^ T cells were then further identified and gated CD4^+^ helper T cells and CD8^+^ cytotoxic T cells.

**B**. CD3^+^ T cells were gated followed by analysis of Annexin- V versus 7-AAD. Annexin- V ^+^ 7-AAD^-^ cells were defined as apoptotic cells, and Annexin- V ^+^ 7-AAD^+^ cells were defined as necrotic cells.

**Supplementary Table 1. Primer sequences for real-time quantitative PCR**

| Gene | Forward primer | Reverse primer |
| --- | --- | --- |
| Pre-miR-155 | 5'-TAATCGTGATAGGGGTTT-3' | 5'-GGAGTCAGTTGGAGGC-3' |
| Dicer | 5'-TGCTATGTCGCCTTGAATGTT-3' | 5'-AATTTCTCGATAGGGGTGGTCTA-3' |
| CCL2 | 5'-CAGCCAGATGCAATCAATGCC-3' | 5'-TGGAATCCTGAACCCACTTCT-3' |
| CCL5 | 5'-CCAGCAGTCGTCTTTGTCAC-3' | 5'-CTCTGGGTTGGCACACACTT-3' |
| CXCL10 | 5'-GTGGCATTCAAGGAGTACCTC-3' | 5'-TGATGGCCTTCGATTCTGGATT-3' |
| IL-10 | 5'-ATGCCCCAAGCTGAGAACCAAGACCCA-3' | 5'-TCTCAAGGGGCTGGGTCAGCTATCCCA-3' |
| IL-13 | 5'-GAGTGTGTTTGTCACCGTTG-3' | 5'-TACTCGTTGGTCGAGAGCTG-3' |
| MMP-1 | 5'-CAGAGATGAAGTCCGGTTTTTC-3' | 5'-GGGGTATCCGTGTAGCACAT-3' |
| MMP-9 | 5'-GAACCAATCTCACCGACAGG-3' | 5'-GCCACCCGAGTGTAACCATA-3' |
| TNF-α | 5'-GAGGCCAAGCCCTGGTATG-3' | 5'-CGGGCCGATTGATCTCAGC-3' |
| IFN-γ | 5'-TCGGTAACTGACTTGAATGTCCA-3' | 5'-TCGCTTCCCTGTTTTAGCTGC-3' |
| IL-2 | 5'-CCTTGCACTTCTGAAGAGATTGA-3' | 5'-ACAGGGCCATCATAAAAGAGGT-3' |
| Gapdh | 5'-GTCTCCTCTGACTTCAACAGCG-3' | 5'-ACCACCCTGTTGCTGTAGCCAA-3' |

**Supplementary Table 2. Information of antibodies**

| **Antibody** | **Provider** | **Application** |
| --- | --- | --- |
| anti-human-Calnexin | Santa Cruz, TX,USA | WB |
| anti-human-CD63 | Novus, CO,USA | WB |
| anti-human-CD81 | Novus, CO,USA | WB |
| anti-human-CD163 | Abcam, Cambridge, UK | WB |
| anti-human-CD206 | Abcam, Cambridge, UK | WB |
| anti-human-PD-L1 | Cell Signaling Technology, MA USA | WB |
| anti-human PD-L1-PE | Biolegend, CA, USA | FCM,FACS |
| anti-human CD14-APC | eBioscience, Frankfurt, Germany | FCM,FACS |
| anti-human Annexin V-FITC | Thermo Fisher Scientific | FCM,FACS |
| 7-AAD | Biolegend, CA, USA | FCM,FACS |
| anti-human CD3-BV421 | Biolegend, CA, USA | FCM,FACS |
| anti-human CD4-APC | Thermo Fisher Scientific | FCM,FACS |
| anti-human CD8-PE | Thermo Fisher Scientific | FCM,FACS |
| anti-mouse CD45-APC | eBioscience, Frankfurt, Germany | FCM,FACS |
| anti-mouse CD11c-FITC | Biolegend, CA, USA | FCM,FACS |
| anti-mouse CD11b-Cy5 | eBioscience, Frankfurt, Germany | FCM,FACS |
| anti-mouse Ly6G-BV421 | Biolegend, CA, USA | FCM,FACS |
| anti-mouse F4-80-PE | eBioscience, Frankfurt, Germany | FCM,FACS |
| anti-mouse Ly6C-BV605 | Biolegend, CA, USA | FCM,FACS |
| anti-mouse CD3-BV421 | Biolegend, CA, USA | FCM,FACS |
| anti-mouse CD4-APC | Thermo Fisher Scientific, MA,USA | FCM,FACS |
| anti-mouse CD8-PE | Thermo Fisher Scientific, MA,USA | FCM,FACS |
| DCFH-DA Redox Probe | G-Biosciences, MO,USA | FCM |
| MitoSOX™ Red | Thermo Fisher Scientific, MA,USA | FCM |
| CD3 ultra-leaf purified antibody | Biolegend, CA, USA | Stimulation |
| CD28 ultra-leaf purified antibody | Biolegend, CA, USA | Stimulation |
| anti-mouse-PDL1 | Bio X cell, USA | Blocking |
| Rat IgG isotype control | Bio X cell, USA | Blocking |
| anti-mouse-F4/80 | Cell Signaling Technology, MA USA | IHC |
| anti-mouse-PDL1 | Cell Signaling Technology, MA USA | IHC |
