# Supplementary material for: Reactive oxygen species reprogram macrophages to suppress antitumor immune response through the exosomal miR-155-5p/PD-L1 pathway
Source: J Exp Clin Cancer Res. 2022 Jan 27;41:41. doi: 10.1186/s13046-022-02244-1 (PMC8793215; doi:10.1186/s13046-022-02244-1)
Supplement: Supplementary file 2 — Additional file 2. [file 13046_2022_2244_MOESM2_ESM.docx]

**Supplementary Methods**

**Generation of human and murine ovarian cancer cell lines stably expressing miR-155-5p**

A2780 human ovarian cancer cells were transfected with a human miR-155-5p expression plasmid (Origene, Rockville, US) or a miR-control plasmid using jetPRIME® Transfection Reagent (Polyplus, New York, USA) according to manufacturer’s instruction. At 24 h post-transfection, cells were cultured in fresh growth medium containing selective agent antibiotic Geneticin (G418, Sigma, Millipore, USA). The same method was used to generate a stable murine ovarian cancer ID8 cell line ectopic expression of miR-155-5p using mouse miR-155-5p expression plasmid (Origene, Rockville, US).

**Transient transfection**

miRNA transfection: the negative control miRNA and MIR152 mimics were from Applied Biosystem. Cells were cultured in 6-well plates to reach 60% confluency, and transfected with 25 nM MIR155 mimic or miR-control using Lipofectamine RNAiMAX reagent (Invitrogen) according to the manufacturer's instruction. Total proteins and RNAs were prepared from the cells 60 to 72 h after the transfection, and were used for subsequent analysis.

RNA interference: nontargeting siRNAs pool control (sicont), siRNA Smartpools against Dicer were purchased from Dharmacon. Cells were cultured in 6-well plates to reach 60% confluency and transfected with 50 nM specific siRNAs or control siRNA using X-tremeGENE reagent (Roche) according to the instructions.

Plasmid transfection: macrophages were transfected with pCMV-entry vector or pCMV-entry-PD-L1 (Origene) using JetPRIME transfection reagent (Polyplus, New York, USA) for 48 h before the downstream applications.

**Immunoblotting**

Cells and exosomes were lysed in RIPA lysis buffer (Thermo Fisher Scientific). Sample loading was normalized according to BCA protein assay kit (Pierce). Samples were subsequently boiled for 5 min with 2 × SDS loading buffer, and resolved on polyacrylamide gels followed by transfer onto 0.45-μm pore-size nitrocellulose membranes using wet electrophoretic transfer. After blocking with 5% (w/v) nonfat dry milk for 1 h at room temperature, the protein blot was incubated overnight at 4 °C with the primary antibodies. The sources of primary antibodies were shown in Suppl. Table 2.

**Wound-healing assay**

THP-1 cells were seeded in the 6-well plates (Corning, NY, USA) and differentiated into macrophages by adding PMA at 100 nM for 24 h. Differentiated macrophages were treated with Exo-con/Exo-NAC or Exo-miR-con/Exo-miR-155 (Exosomes isolated from A2780-miR-con/A2780-miR-155 stable cell lines) for 48 h. The medium was then replaced and cells were cultured for another 24 h after scratches were generated using small pipette tips. The images were photographed at 0 and 24 h with light microscopy (Olympus CKX31). The gap size and its rate of closure over time was measured and analyzed using Image J software.

**Electron microscopy**

Purified exosomes were prepared as described above. For electron microscopy, exosomes were fixed with 2% paraformaldehyde, loaded on carbon film grids, then placed on 2% gelatin, rinsed with water and then blocked using 1% gelatin from cold water fish skin . The samples were then analyzed with electron microscopy and photographed using an AMT CCD camera.

**ROS detection**

Briefly, cells were incubated in serum-free medium for 16 h, and then were collected in tubes in PBS with 5 μM DCFH-DA (for H_2_O_2_, NAC, catalase-PEG treatments) or MitoSOX^TM^ Red (Thermo Fisher Scientific) (for rotenone treatment) for 30 min followed by flow cytometry analysis. For CellROX orange staining, cells were incubated in serum-free medium for 16 h and then loaded with CellROX orange for 45 min. The samples were washed with PBS, and fixed with 10% buffered formalin for 10 min. All images were collected with an inverted fluorescent microscope with the same camera exposure time. Cell fluorescent intensity was measured using ImageJ software.

The samples

were washed, and intracellular ﬂuorescence was imaged

using an Olympus IX83 Inverted Microscope

The samples

were washed, and intracellular ﬂuorescence was imaged

using an Olympus IX83 Inverted Microscope
